# Mechanism of the *Escherichia coli* MltE lytic transglycosylase, the cell-wall-penetrating enzyme for Type VI secretion system assembly

**DOI:** 10.1038/s41598-018-22527-y

**Published:** 2018-03-07

**Authors:** Byungjin Byun, Kiran V. Mahasenan, David A. Dik, Daniel R. Marous, Enrico Speri, Malika Kumarasiri, Jed F. Fisher, Juan A. Hermoso, Shahriar Mobashery

**Affiliations:** 1 Department of Chemistry and Biochemistry, University of Notre Dame, Notre Dame, Indiana, 46556 United States; 2 Department of Crystallography and Structural Biology, Instituto de Química-Física “Rocasolano”, Consejo Superior de Investigaciones Científicas, 28006 Madrid, Spain

## Abstract

Lytic transglycosylases (LTs) catalyze the non-hydrolytic cleavage of the bacterial cell wall by an intramolecular transacetalization reaction. This reaction is critically and broadly important in modifications of the bacterial cell wall in the course of its biosynthesis, recycling, manifestation of virulence, insertion of structural entities such as the flagellum and the pili, among others. The first QM/MM analysis of the mechanism of reaction of an LT, that for the *Escherichia coli* MltE, is undertaken. The study reveals a conformational itinerary consistent with an oxocarbenium-like transition state, characterized by a pivotal role for the active-site glutamic acid in proton transfer. Notably, an oxazolinium intermediate, as a potential intermediate, is absent. Rather, substrate-assisted catalysis is observed through a favorable dipole provided by the *N*-acetyl carbonyl group of MurNAc saccharide. This interaction stabilizes the incipient positive charge development in the transition state. This mechanism coincides with near-synchronous acetal cleavage and acetal formation.

## Introduction

**Figure 1:**
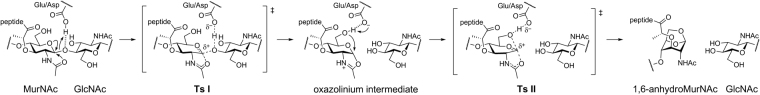
Proposed mechanism for lytic transglycosylases. The catalytic Glu/Asp acts initially as a general acid, donating its proton to the glycosidic oxygen of the scissile bond. The developing positive charge in the oxocarbenium transition state (**Ts I**) may be stabilized through the formation of a putative oxazolinium intermediate involving the *N*-acetyl group of −1 MurNAc. The deprotonated glutamate/aspartate then acts as a general base to activate the C6-OH for intramolecular attack at the anomeric carbon. This process collapses the oxazolinium intermediate with the concomitant formation of the 1,6-anhydroMurNAc reaction product, having all of its substituents in an axial orientation.

The lysozyme family of the glycoside hydrolases (GHs) catalyzes the cleavage of the β-1 → 4-glycosidic linkage connecting the *N*-acetylmuramic acid (MurNAc) and *N*-acetyl-d-glucosamine (GlcNAc) saccharides of the (MurNAc-GlcNAc)_n_ polymer (the peptidoglycan) of the cell wall of bacteria. While the non-bacterial lysozymes themselves are hydrolytic catalysts, the lytic transglycosylase (LT) sub-families of the GHs are not^1,2^. The LTs act on the same MurNAc-β-1 → 4-GlcNAc glycosidic linkage of the peptidoglycan to accomplish a non-hydrolytic scission so as to create two daughter strands having (respectively) 1,6-anhydroMurNAc and GlcNAc termini (Fig. 1)^3–5^. LT catalysis is used by bacteria for a host of functional transformations, including peptidoglycan biosynthesis, remodeling, recycling, and excavation for insertion of secretion systems and of flagella and pili. Evidence also correlates LT dysregulation to the bactericidal mechanism of the β-lactam antibiotics^6^. The range of cell-wall processes that the LT reactions enable is impressive. This first QM/MM analysis of the LT reaction was undertaken to shed light on this unique transformation, critical for homeostasis of the bacterial cell wall.

The stereochemistry of LT catalysis is overall retention with respect to the anomeric carbon of the MurNAc saccharide. While the origin of the anhydroMurNAc product is that of an intramolecular interception of an oxocarbenium entity, the steps leading to this event are uncertain. One proposed mechanism uses substrate-assistance by forming an oxazolinium intermediate. In this mechanism, the MurNAc amide functional group acts first as a nucleophile and then as a nucleofuge (Fig. 1)^7^. Oxazolinium intermediates in GlcNAc glycosyl transfer are well recognized^8^, and invoking this intermediate would account for the retention of configuration by the LTs through a sequence of two half-reactions, each requiring inversion^9^. This proposed intermediate was also suggested to account for dispersing the charge developed at the anomeric carbon during bond breaking^10^.

Within the LT sub-family, a glutamic (or aspartic) acid has the pivotal catalytic role. We elected to use QM/MM analysis to elucidate the LT reaction mechanism, using the membrane-bound lytic transglycosylase E (MltE) enzyme of *Escherichia coli* as our example. The small mass of this protein (approximately 21 kDa), the absence of peripheral domains that may impart influence, and the availability of quality crystal structures for MltE made it the appropriate choice for this study. MltE is a lipoprotein catalyst involved in the late stages of type VI secretion system assembly^11^. MltE is also the primary endolytic LT (*i*.*e*., cleavage in the middle of a peptidoglycan strand) of *E*. *coli*^12,13^. Its QM/MM study was anticipated to provide the first insights into the enigmatic mechanism of the LT enzymes.

**Figure 2:**
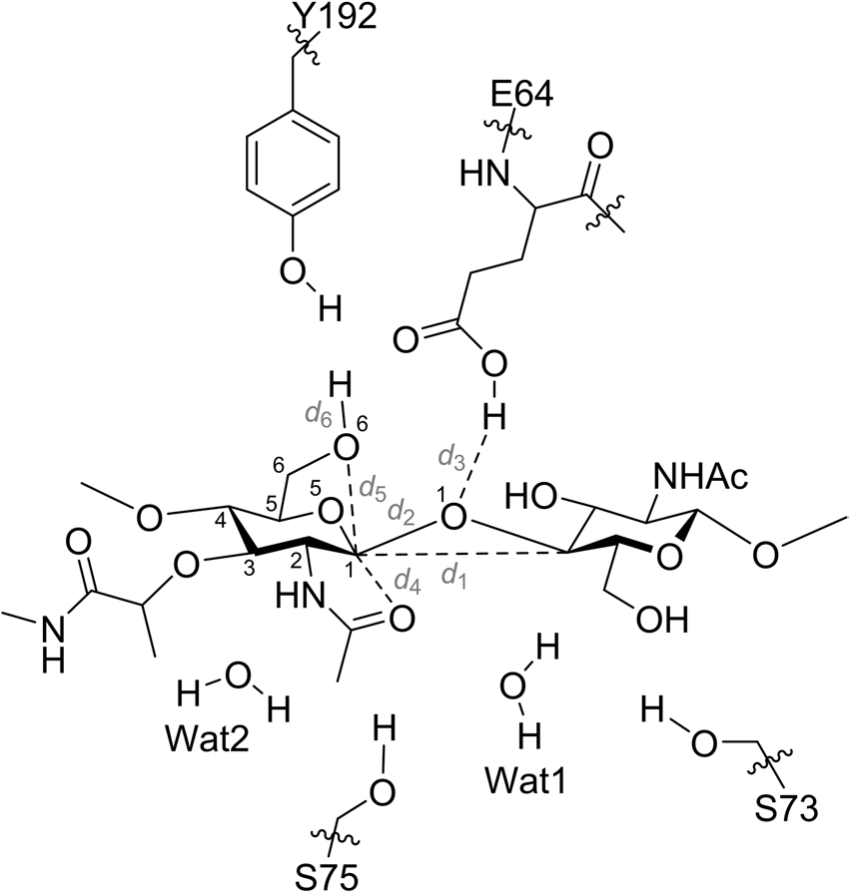
Atoms included in the QM layer and the reaction parameters. The QM layer includes 123 atoms: the MurNAc-GlcNAc substrate; residue E64; the side chains of S73, S75, and Y192; and the two active-site water molecules (Wat1 and Wat2).

**Figure 3:**
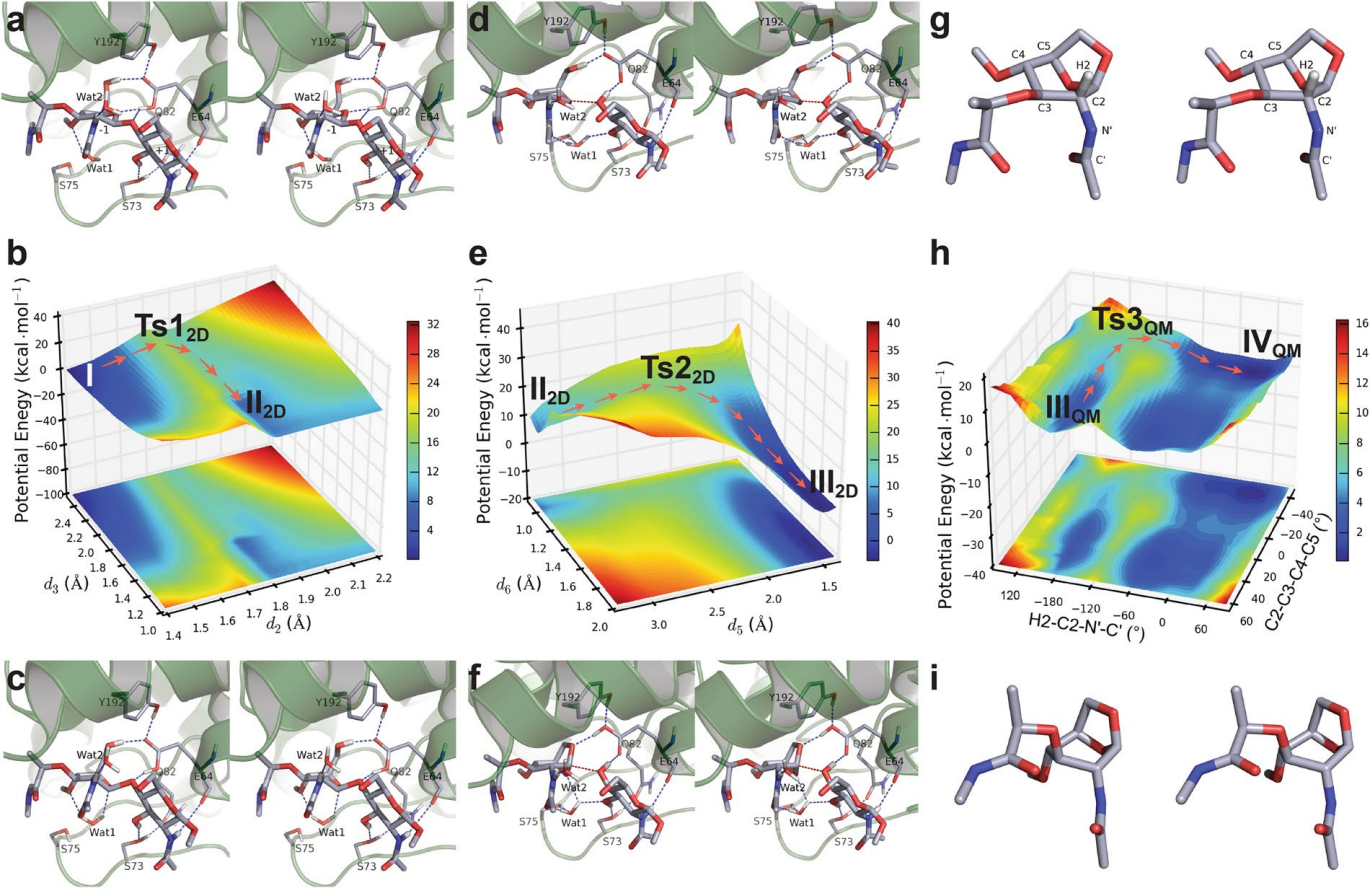
MltE transformation. (**a**) Stereo representation of the Michaelis complex **I**. (**b**) The potential-energy surface with respect to the *d*_2_ and *d*_3_ reaction coordinates. (**c**) Intermediate **II**_**2D**_. (**d**) Transition species **Ts2**_**2D**_ between **II**_**2D**_ and **III**_**2D**_. (**e**) The reaction path (orange arrows) from **II**_**2D**_ through **Ts2**_**2D**_ to **III**_**2D**_. (**f**,**g**) 1,6-anhydroMurNAc in the *B*_3,O_ conformation (**III**_**2D**_ and **III**_**QM**_). (**h**) The QM potential-energy surface for *B*_3,O_ to ^1^*C*_4_ conformational change. (i) 1,6-anhydroMurNAc in the ^1^*C*_4_ conformation (**IV**_**QM**_). Hydrogen bonds and the *d*_2_ are shown as blue and red dashed lines, respectively.

**Figure 4:**
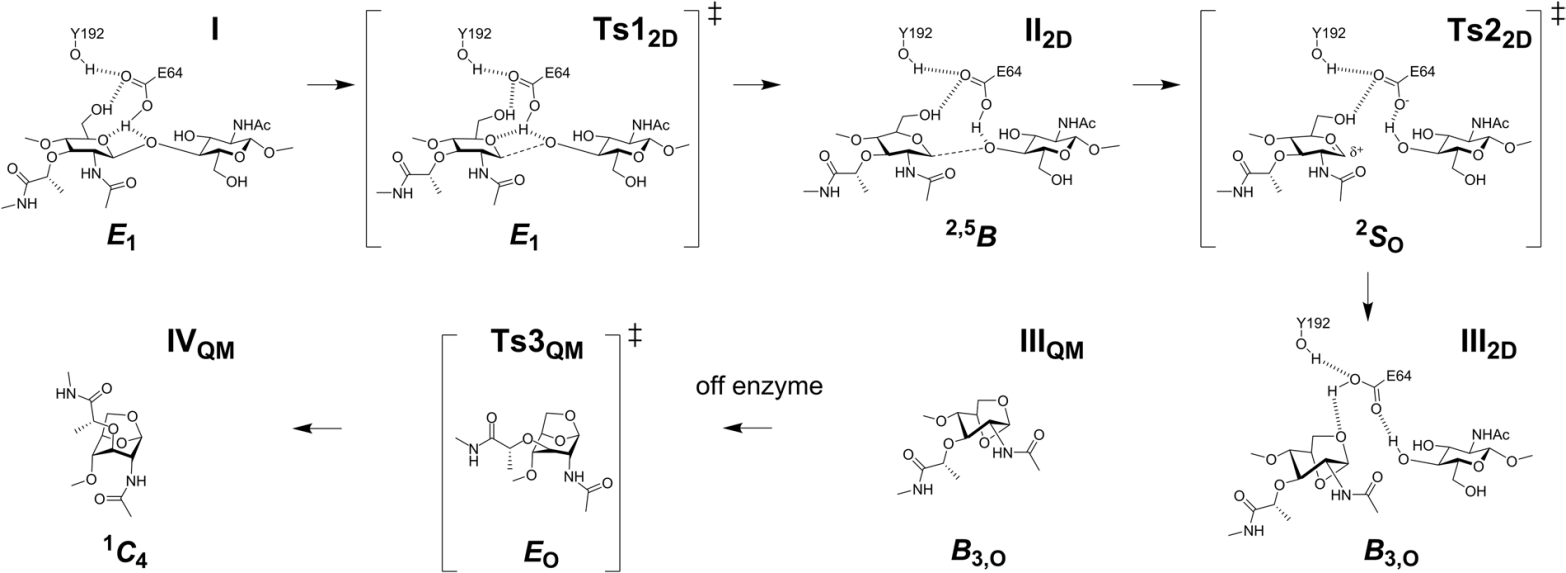
Conformational itinerary of −1 MurNAc along MltE transformation. **I** denotes the Michaelis complex; **Ts1**_**2D**_, the transition species between **I** and **II**_**2D**_; **II**_**2D**_, the intermediate; **Ts2**_**2D**_, the transition species between **II**_**2D**_ and **III**_**2D**_; **III**_**2D**_ and **III**_**QM**_, the 1,6-anhydroMurNAc in the *B*_3,O_ conformation; **Ts3**_**QM**_, the transition species between **III**_**QM**_ and **IV**_**QM**_; **IV**_**QM**_, the 1,6-anhydroMurNAc in the ^1^*C*_4_ conformation. A −1 MurNAc adopts boat (*B*), chair (*C*), envelope (*E*), and skew (*S*) conformations. The *B*_3,O_ to ^1^*C*_4_ transformation is off-enzyme reaction.

We analyzed the molecular-dynamics production-phase trajectory of the complex and selected several snapshots that had suitable distances (*d*_3_, *d*_4_, and *d*_5_) for the proton transfer events (Fig. 2 and Supplementary Fig. 1). One of the snapshots with appropriate distance parameters after MM optimizations was selected for the QM/MM calculations (see Supplementary Computational Methods for details). The selection of residues for the QM layer (Fig. 2) was made with attention to a comparative sequence analysis of the LT family and available X-ray structures (Supplementary Figs 2–5). Residue E64 (the catalytic glutamic acid); the side chains of S73, S75, and Y192; and the two active-site water molecules (Wat1 and Wat2) were carefully selected. The Michaelis complex (Fig. 3a and Supplementary Fig. 6) was obtained following two-layer ONIOM^14^ QM/MM energy minimization method. The dominant features of the Michaelis complex are the hydrogen-bonding pattern of E64 and the *E*_1_ conformation of the −1 MurNAc saccharide (see Supplementary Information for details: the hexose conformers are described as boat (*B*), chair (*C*), envelope (*E*, previously “sofa”), half-chair (*H*, sometimes called half-boat or twist), and skew (*S*, sometimes called twist-boat) conformations (Fig. 4), according to the Cremer and Pople nomenclature)^15–17^. The competence of this Michaelis complex was tested for its ability to traverse the full reaction coordinate across a 1D potential-energy surface (PES) scan (see Supplementary Information for details). Coordinates obtained from the 1D PES provided the requisite starting points for two subsequent 2D-PES calculations of the key reaction steps. The first 2D-PES scan corresponds to the formation of the local energy minimum (**II**_**2D**_), and the second scan to the formation of 1,6-anhydroMurNAc in a *B*_3,O_ conformation (**III**_**2D**_) (Fig. 3).

The first 2D-PES scan starts from the Michaelis complex (**I**) and uses the glycosidic bond (*d*_2_, scanned at 0.10 Å intervals from 1.40 to 2.20 Å) and the distance between the glycosidic oxygen (O1) and the O^ε2^ hydrogen (H^ε2^) of E64 (*d*_3_, scanned at –0.10 Å intervals from 2.50 to 1.00 Å) as the reaction coordinates. The lengthening of the C–O glycosidic bond is accompanied by the approach of hydrogen atom from E64 to O1. In this progression, the MurNAc undergoes an *E*_1_ → [*E*_1_]^⧧^ → ^2,5^*B* conformational path. The locations of the Michaelis complex **I** (Fig. 3a and Supplementary Fig. 6: *d*_2_ = 1.40 Å, *d*_3_ = 2.50 Å) and the local minimum **II**_**2D**_ (Fig. 3c and Supplementary Fig. 7: *d*_2_ = 1.80 Å, *d*_3_ = 1.50 Å, see Supplementary Information for details) are indicated on the 2D PES (Fig. 3b). The arrows show the progression from **I** to **II**_**2D**_. The energy of **II**_**2D**_ is 6.55 kcal·mol^−1^ higher than that of **I**. Transition point 1 (Supplementary Fig. 8: species **Ts1**_**2D**_ at *d*_2_ = 1.70 Å and *d*_3_ = 2.40 Å) retains the *E*_1_ conformation and is 17.09 kcal·mol^−1^ higher in energy than **I**.

Species **II**_**2D**_ progresses toward the transition species **Ts2**_**2D**_, having the fully broken (2.90 Å) glycosidic bond in our second 2D-PES calculation. Continued progression results in the formation of 1,6-anhydroMurNAc in a *B*_3,O_ conformation (species **III**_**2D**_), as shown in Fig. 3. Proton transfer mediated by the E64 carboxylate acting as a general base directs intramolecular bond formation between the O6 and the anomeric C1 of MurNAc. The reaction coordinates *d*_5_ (distance between O6 and C1 of MurNAc, corresponding to bond formation) and *d*_6_ (O6–H bond of MurNAc, corresponding to proton transfer) define the path to formation of species **III**_**2D**_ (Fig. 2). These distances were scanned at 0.10 Å intervals (*d*_5_ from 3.29 to 1.39 Å, and *d*_6_ from 0.90 to 2.00 Å). The resulting PES (Fig. 3e) shows the second transition species in a ^2^*S*_O_ skew conformation (Fig. 3d: species **Ts2**_**2D**_ at *d*_5_ = 2.29 Å and *d*_6_ = 1.00 Å) and the 1,6-anhydroMurNAc in a *B*_3,O_ conformation (Fig. 3f: species **III**_**2D**_ at *d*_5_ = 1.49 Å and *d*_6_ = 1.80 Å). Progression along this path (arrows) to species **III**_**2D**_ at −3.91 kcal·mol^−1^ is exothermic with respect to the Michaelis complex. The transition species **Ts2**_**2D**_, between species **II**_**2D**_ and **III**_**2D**_, is 10.39 kcal·mol^−1^ higher in potential energy than **II**_**2D**_.

The progress from **II**_**2D**_ to **Ts2**_**2D**_ coincides with an increased positive charge on C1 (from 0.451 to 0.522 *e*), as calculated by natural population atomic charges (Supplementary Table 1). During this progress, the distance between the *N*-acetyl carbonyl oxygen of MurNAc (O_NAc_) and the C1 shortens in order to stabilize the incipient positive charge on C1. As *d*_2_ increases from 1.80 to 2.90 Å, *d*_4_ (the distance between C1 and O_NAc_) decreases from 3.14 to 2.83 Å. The increase in *d*_2_ provides the necessary space between C1 and O1 for the H1 (the hydrogen on the C1) to assume the planar arrangement for the oxocarbenium species in **Ts2**_**2D**_. The MurNAc of **Ts2**_**2D**_ shows a dihedral angle of C5–O5–C1–C2 (37.4°) and an out-of-plane angle of *θ*_H1_ (10.9°). Additionally, the distance between C1–O5 shortens from 1.35 to 1.28 Å. These changes reflect the oxocarbenium character of species **Ts2**_**2D**_.

Species **Ts2**_**2D**_ has a ^2^*S*_O_ conformation, indicating a ^2,5^*B* → [^2^*S*_O_]^⧧^ → *B*_3,O_ conformational path. The ^2^*S*_O_ conformation provides a favorable orientation for the in-line approach of O6 to C1. The O6 of MurNAc has more negative charge than the O_NAc_ (–0.700 *e* and –0.619 *e*, respectively). At the same time, the distance of C1 to O6 (2.29 Å) is less than that of C1 to O_NAc_ (2.83 Å). The suitably positioned (now serving as a general base) E64 O^ε1^ activates the C6 hydroxyl by proton abstraction. Interception of the oxocarbenium by O6 transforms **Ts2**_**2D**_ to the 1,6-anhydroMurNAc product in a *B*_3,O_ conformation (**III**_**2D**_).

We note the importance of solvation within the active site for catalysis. In the **Ts2**_**2D**_ species, Wat1 bridges between the H^γ^ of S75 and the O_NAc_ via hydrogen bonds (1.83 Å for both, Fig. 3d). In addition, Wat1 forms another hydrogen bond (1.89 Å) with the oxygen of C3 hydroxyl group of GlcNAc. These hydrogen bonds are maintained throughout the transition from species **II**_**2D**_ to **Ts2**_**2D**_. The location of Wat1 prevents oxazolinium formation. Interestingly, a water molecule poised in a similar location is observed in the X-ray co-crystal structures (PDB IDs: 4HJZ, 1QTE, 1QUT, 1D0K, 5AO7, 3D3D, and 1D9U) of LT enzymes MltE, Slt70, Slt35, SltB3, and bacteriophage endolysin lambda^18–23^. The failure to observe formation of an oxazolinium intermediate is attributable in part to the proximity of C1 to O1 (a short distance of 1.80 Å). This proximity prevents the H1 from achieving planarity. In addition, PES calculated after computational deletion of Wat1 from the QM layer gave **Ts1** at a value of 6.8 kcal·mol^−1^ higher than that with Wat1 present (23.9 kcal·mol^−1^ vs 17.1 kcal·mol^−1^ respectively; Supplementary Fig. 9). Hence, conservation of this active-site water molecule is not merely structural, but also contributes to transition-state stabilization.

**Figure 5:**
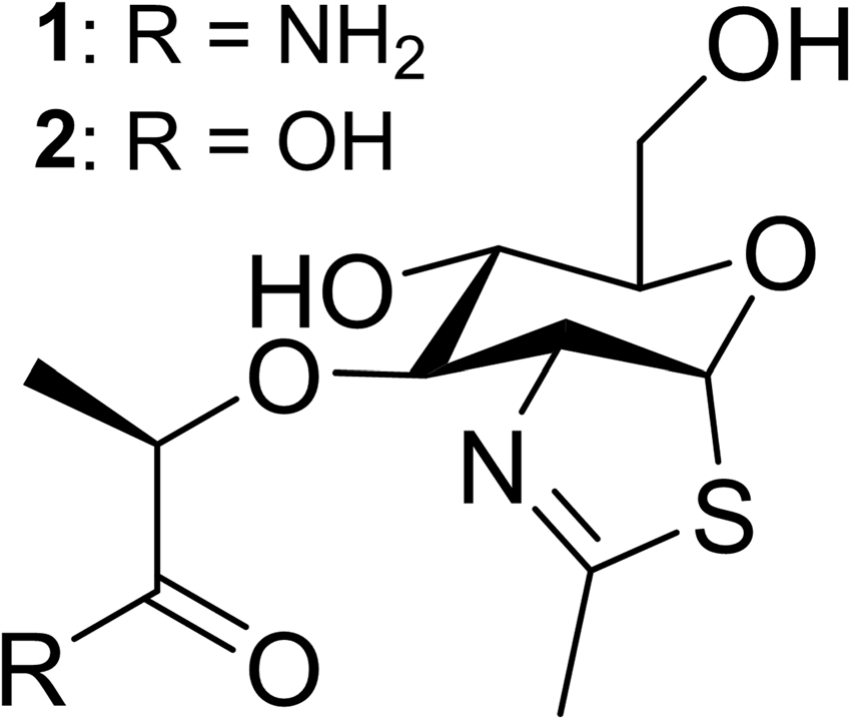
Chemical structures of compounds **1** and **2**. The compounds **1** and **2** are MurNAc-based thiazoline derivatives, analogs of the putative oxazolinium intermediate (see Fig. 1).

The absence of the oxazolinium species from the two PESs likely is linked with the lack of a second carboxylic acid in the MltE active site. In retaining GH enzymes (GH18, 20, 25, 56, 84, 85, and 123) where this second carboxylate is present, the energetic demand for formation of an oxocarbenium intermediate is mitigated by its interception by the proximal acetamide, to form an oxazolinium intermediate^8,24–31^. In our computational study, however, the oxazolinium is not a local energy minimum. A 2D PES (Supplementary Fig. 10) generated by scanning *d*_4_ (the distance between C1 and O_NAc_) and *d*_5_ (the distance between O6 and C1 of MurNAc) as the coordinates further interrogated its structure. We examined the PES to verify that the dipole interaction with O_NAc_ contributes greater stability to the charge development on C1 compared to the bonding represented by an oxazolinium cation (defined by a *d*_4_ value of 1.58 Å). None of the species with *d*_4_ = 1.58 Å was more stable than species with *d*_4_ > 1.58 Å. Formation of the oxazolinium in the absence of energetic compensation by the second catalytic residue is unfavorable due to the charge development on *N*-acetyl nitrogen of MurNAc. The water molecules, including Wat2, cannot provide sufficient stabilizing effect for the charge development on the nitrogen of the 2-acetamido group of the oxazolinium. We followed up the observation from the calculation with experiments. The oxazolinium moiety is relatively unstable in solution, however the thiazoline (thiazolinium) analog is stable, and its inhibitory activity against GH enzymes that form an oxazolinium intermediate is regarded as a diagnostic of this mechanistic pathway^32^. We synthesized two MurNAc-based thiazoline derivatives (compounds **1** and **2**, Fig. 5). If the oxazolinium species were an enzyme intermediate, one would expect that thiazolines **1** or **2** would provide some degree of inhibition of MltE. At concentrations as high as 1 mM, we observed no inhibition of MltE by either compound (Supplementary Fig. 11).

The final step of the reaction is a boat-to-chair transformation. The ground-state conformation of 1,6-anhydroMurNAc is a ^1^*C*_4_ chair. Before 2D-PES calculations, the relaxation of the *B*_3,O_ conformation of **III**_**2D**_ to the ^1^*C*_4_ conformation first was assessed by a 1D-PES scan of the MurNAc C2–C3–C4–C5 dihedral angle of species **III**_**2D**_. During this QM/MM scan, the energy of the system increased continuously and the potential-energy surface failed to give a local energy minimum. This progressive increase in energy results from a steric clash between the C3 lactyl moiety and the protein surface. The *B*_3,O_-to-^1^*C*_4_ transformation cannot occur within the active site, but must occur during the course of (or subsequent to) release of the 1,6-anhydroMurNAc product. Indeed, the favorable energy change of this transformation might be a critical driving force for product release. To gain insight into the energy barrier for this boat-to-chair transformation, a QM 2D-PES scan was conducted for the 1,6-anhydroMurNAc outside of the active site. Two dihedral angles were scanned in this calculation; dihedral C2–C3–C4–C5 (from 63.9 to −46.1° with −5.0° intervals) and dihedral H2–C2–N′–C′ (from −180.0 to 150.0° with 30.0° intervals). The 2D PES shows a potential energy for the *B*_3,O_ boat that is +2.00 kcal·mol^−1^ above the ^1^*C*_4_ chair (Fig. 3h). The transition point (Supplementary Fig. 12: species **Ts3**_**QM**_ at C2–C3–C4–C5 = −31.1° and H2–C2–N′–C′ = −150.0°) adopts an *E*_O_ conformation that is +5.50 kcal·mol^−1^ higher than the *B*_3,O_ conformation. Interestingly, concomitant rotation occurs about the C2–N′ single bond, with the boat-to-chair transition. This rotation can be attributed to a relieving of the electrostatic repulsions in the ^1^*C*_4_ conformation among the O5, C4 oxygen, and *N*-acetyl carbonyl oxygen.

**Figure 6:**
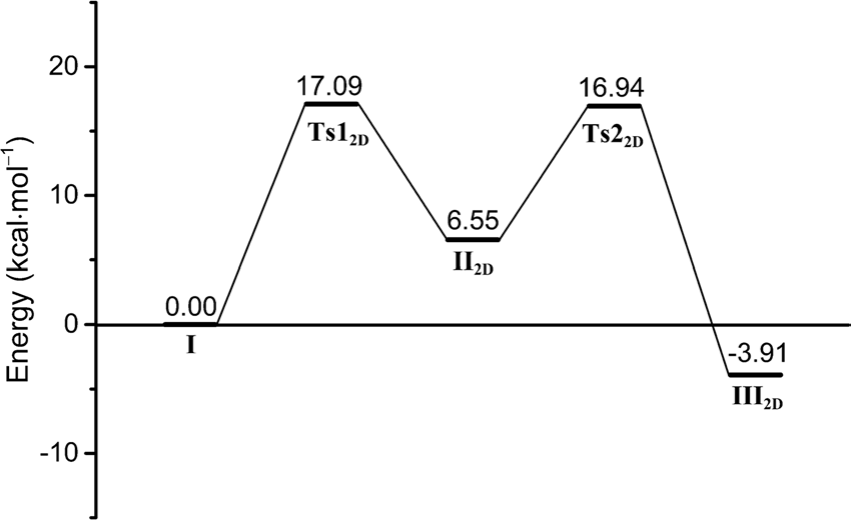
Potential-energy profiles for MltE transformations. **I** denotes the complex of the substrate bound into the MltE active site; **Ts1**_**2D**_, the transition point between species **I** and **II**_**2D**_; **II**_**2D**_, the intermediate; **Ts2**_**2D**_, the transition point between species **II**_**2D**_ and **III**_**2D**_; **III**_**2D**_, the 1,6-anhydroMurNAc in the ***B***_3,O_ conformation.

GHs bind their carbohydrate substrates in the non-ground-state conformation that optimally positions the exocyclic moiety at the anomeric carbon for departure as a leaving group. Understanding this conformational distortion is recognized as having widespread value for the development of GH inhibitors as antibiotics or potentiators of clinical antibiotics^33^. Our calculations support existence of such a substrate distortion in MltE. The conformation of the MurNAc in the peptidoglycan in solution is ^1^*C*_4_. On binding to MltE, overall turnover chemistry uses an *E*_1_ → [*E*_1_]^⧧^ → ^2,5^*B* → [^2^*S*_O_]^⧧^ → *B*_3,O_ → [*E*_O_]^⧧^ → ^1^*C*_4_ conformational itinerary (Figs 4 and 6). The initial *E*_1_ conformation imposed by MltE on its MurNAc substrate enables access to the transition point 1 (**Ts1**_**2D**_) through least motion of the nuclei^33^. Formation of the intermediate **II**_**2D**_, by lengthening of the C–O glycosidic bond in response to hydrogen bonding by E64 (activation barrier of 17.09 kcal·mol^−1^) is overall endothermic by 6.55 kcal·mol^−1^. Subsequent further glycosidic-bond lengthening and proton transfer gives a free-base E64 poised to activate the C6 hydroxyl for interception of the oxocarbenium (**Ts2**_**2D**_). The ^2^*S*_O_ conformation for **Ts2**_**2D**_ provides a favorable in-line approach of the nucleophilic O6 oxygen. Intramolecular bond formation between O6 and C1 gives the 1,6-anhydroMurNAc product in a *B*_3,O_ conformation (species **III**_**2D**_). The transition species **Ts2**_**2D**_ is 10.39 kcal·mol^−1^ higher in potential energy relative to **II**_**2D**_, and formation of **III**_**2D**_ is exothermic by −3.91 kcal·mol^−1^. There does not appear to be a unique rate-limiting step, as the two transition-step species are essentially of equal energy (Fig. 6). Relaxation of the *B*_3,O_ boat to the ^1^*C*_4_ 1,6-anhydroMurNAc chair is concurrent with, or subsequent to, product release. Our calculations are consistent with a mechanism of near-synchronous bond formation and bond cleavage, enabled by complementary conformational and electrostatic stabilization. In this particular aspect, the reaction of MltE is an example of the mechanistically challenging front-face retaining glycoside-transferase enzymes^9^, exhibiting a near-synchronous pathway involving a short-lived oxocarbenium-like species^34^.

The LT family is implicated in a host of transformations preserving the function and integrity of the bacterial cell wall. This study establishes a mechanistic framework for further interrogation of the critical roles this family has in the biosynthesis, maturation and turnover of this important biopolymer.

## Methods

### Calculations

Molecular dynamics (MD) simulations used the AMBER 11 suite^35^. AMBER FF99 and GAFF provided simulation parameters. A snapshot was selected as the starting point, chosen by monitoring the distances and angles from the production phase trajectory, for the design of the QM/MM calculation. The two-layer version of the ONIOM^14^ method implemented in Gaussian 09^36^ was used. In a two-layer ONIOM method, the total energy of the system is obtained from three independent calculations: *E*_ONIOM_ = *E*_real,MM_ + *E*_model,QM_ – *E*_model,MM_, where ‘real’ refer to the whole system and ‘model’ refers to the chemically important part of the system (QM layer). The real system is calculated at MM level. MM method cannot describe bond breaking or formation. The model system is treated with more accurate, but considerably expensive QM method. The QM layer used the B3LYP/6-311++G(d,p)//B3LYP/6-31 G(d) level of theory while the MM layer used the AMBER FF99 force field. The QM layer included 123 atoms: the MurNAc-GlcNAc substrate; the catalytic residue E64; the side chains of S73, S75, and Y192; and the two active-site water molecules (Wat1 and Wat2). Potential-energy points in the QM/MM calculations were generated over a two-dimensional grid of two direct coordinates. All the stationary points (*i*.*e*. species **I**, **Ts1**_**2D**_, **II**_**2D**_, **Ts2**_**2D**_, **III**_**2D**_, **III**_**QM**_, **Ts3**_**QM**_, and **IV**_**QM**_) were fully optimized with no reaction coordinate constraints before characterization by frequency calculations. Frequency calculations were performed with scale factors of 0.873 and 0.944 at B3LYP and M06-2X levels of theory, respectively, at 25 °C and 1 atm. For more details, see Supplementary Computational Methods.

### (2*R*)-2-[[(3a*R*,5*R*,6*S*,7*R*,7a*R*)-3a,6,7,7a-tetrahydro-6-hydroxy-5-(hydroxymethyl)-2-methyl-5*H*-pyrano[3,2-d]thiazol-7-yl]oxy]propanamide (1)

Compound **6** (0.10 g, 0.32 mmol, see Supplementary Information) was dissolved in methanol (4 mL) and 7 N ammonia in methanol was added (3 mL, 21 mmol). The reaction mixture was stirred overnight at RT, similar to a previously reported method)^37^. The reaction mixture was filtered through a cotton plug and concentrated by rotary evaporation. The titled compound was obtained as a white solid (97 mg, 99%) after vacuum drying: TLC (1:9 MeOH:CH_2_Cl_2_): *R*_f_ = 0.17. ^1^H NMR (400 MHz, CD_3_OD) δ 1.43 (d, *J* = 6.9 Hz, 3 H), 2.27 (d, *J* = 2.0 Hz, 3 H) 3.37 (ddd, *J* = 11.3, 5.6, 2.7 Hz, 1 H), 3.63–3.69 (m, 1 H), 3.69–3.73 (m, 1 H), 3.73–3.80 (m, 1 H), 3.91 (t, *J* = 4.8 Hz, 1 H), 4.31 (q, *J* = 6.9 Hz, 1 H), 4.48 (dddq, *J* approx 7.1, 5.1, 1.0, 2.0 Hz, 1 H), 6.38 (d, *J* = 7.1 Hz, 1 H); ^13^C NMR (101 MHz, CD_3_OD) δ 18.16, 19.38, 61.51, 68.45, 74.95, 75.76, 77.34, 80.44, 89.48, 169.55, 177.90. MS (*m/z*): [M + H]^+^, calcd for C_11_H_19_N_2_O_5_S, 291.1009; found, 291.1023.

### (2*R*)-2-[[(3a*R*,5*R*,6*S*,7*R*,7a*R*)-3a,6,7,7a-tetrahydro-6-hydroxy-5-(hydroxymethyl)-2-methyl-5*H*-pyrano[3,2-d]thiazol-7-yl]oxy]propanoic acid (2)

Compound **6** (0.10 g, 0.32 mmol) was dissolved in 1:1 THF:water (2 mL). Solid LiOH monohydrate (15 mg, 0.36 mmol) was added. The mixture was stirred for 2 h at RT. The solution was filtered using a cotton plug and concentrated. The solid was vacuum dried to give (100 mg, 99%) of an off-white solid: ^1^H NMR (400 MHz, CD_3_OD) δ 1.35 (d, *J* = 6.9 Hz, 3 H), 2.25 (d, *J* = 2.5 Hz, 3 H), 3.08–3.21 (m, 1 H), 3.54 (dd, *J* = 12.0, 6.4 Hz, 1 H), 3.63–3.76 (m, 2 H), 4.04–4.19 (m, 2 H), 4.67 (dddq, *J* approx. 7.1, 5.1, 1.0, 2.0 Hz, 1 H), 6.34 (d, *J* = 7.1 Hz, 1 H); ^13^C NMR (101 MHz, CD_3_OD) δ 18.61, 18.80, 62.49, 68.16, 73.98, 76.12, 77.76, 78.80, 88.84, 168.73, 180.25. MS (*m/z*): [M + H]^+^, calcd for C_11_H_17_LiNO_6_S, 298.0931; found, 298.0915.

### Cloning and purification of MltE wild-type

The cloning and purification of MltE from *E*. *coli* K12 substrain MG1655 was previously reported by our lab^38^. MltE wild-type was cloned into pET-24a(+) vector (Novagen) using restriction enzyme NdeI to XhoI. The gene encodes for residues 19–203 of MltE, an N-terminal methionine, and a non-cleavable C-terminal LEHHHHHH (membrane anchor and signal peptide removed; residues 1–18 of MltE). The wild-type MltE was expressed and purified as previously reported. The final concentration of the MltE wild-type was determined by a BCA (Bicinchoninic Acid) Protein Assay kit (Pierce). The final yield of the purification was approximately 56 mg of protein per 0.5 L of liquid culture. The proteins were stored at –80 °C and after thawing on ice, no precipitate formed.

### Lytic transglycosylase activity assay

The *E*. *coli* MltE fluorescence activity assays were conducted using a EnzChek® Lysozyme Assay Kit (Invitrogen). The kit includes fluorescein-labeled sacculus (cell wall) from the Gram-positive bacteria *Micrococcus lysodeikticus*. Sacculus of *Micrococcus* species is commonly used in the analysis of LT activity, as it is commercially available and provides a high-level of reactivity with LTs^18^. MltE reactions (100 μL) were prepared by incubation of 50 μL of succulus (substrate at a 1X dilution in 100 μL, as described in the kit) and 50 μL of MltE (final protein concentration 8 μM). Immediately after mixing, the change in fluorescent intensity was monitored for 30 minutes at room temperature on a Cary Eclipse Fluorescence Spectrophotometer (Agilent). Prior to the experiment, the protein was buffer exchanged into 100 mM NaPO_4_, pH 7.5 supplemented with 100 mM NaCl using a Zeba Desalting Column (Thermo Fisher Scientific). Reactions containing compound **1** or **2** were incubated on ice for 20 min in the presence of MltE, prior to incubation with the sacculus at the start of the reaction. Fluorescence readings were obtained at an excitation wavelength of 485 nm and an emission wavelength of 516 nm. The results are displayed in Supplementary Fig. 11.

## Electronic supplementary material

Supplemental Information
