# Supplementary material for: Mechanism of the Escherichia coli MltE lytic transglycosylase, the cell-wall-penetrating enzyme for Type VI secretion system assembly
Source: Sci Rep. 2018 Mar 7;8:4110. doi: 10.1038/s41598-018-22527-y (PMC5841429; doi:10.1038/s41598-018-22527-y)
Supplement: Supplementary file 1 — Supplemental Information [file 41598_2018_22527_MOESM1_ESM.pdf]

# Supplementary Information

# Mechanism of the *Escherichia coli* MltE lytic transglycosylase, the cell-wall-penetrating enzyme for Type VI secretion system assembly

Byungjin Byun[1], Kiran V. Mahasenan[1], David A. Dik[1], Daniel R. Marous[1], Enrico Speri[1], Malika Kumarasiri[1], Jed. F. Fisher[1], Juan A. Hermoso[2] & Shahriar Mobashery[1,*]

[1]Department of Chemistry and Biochemistry, University of Notre Dame, Notre Dame, Indiana 46556, United States
[2]Department of Crystallography and Structural Biology, Instituto de Química-Física "Rocasolano", Consejo Superior de Investigaciones Científicas, 28006 Madrid, Spain

## Table of contents

Computational methods 3
Supplementary Figure 1 4
Supplementary Figure 2 5
Supplementary Figure 3 6
Sequence analysis 7
Supplementary Figure 4 8
Supplementary Figure 5 9
Michaelis complex 10
Supplementary Figure 6 10
1D potential–energy surface 11
Characterization of species $\mathbf{II_{2D}}$ 12
Supplementary Figure 7 12
Supplementary Figure 8 13
Supplementary Figure 9 14
Supplementary Figure 10 15
Supplementary Figure 11 16
Supplementary Figure 12 17
Supplementary Table 1 18
Synthesis of compounds 19
NMR 21
References 26

## Supplementary Computational Methods

MD simulations used the AMBER 11 suite[1]. A MurNAc-GlcNAc disaccharide substrate was built into the active site of apo MltE (PDB ID: 2Y8P, 2.0 Å resolution) using the chitopentose·MltE (PDB ID: 4HJZ, 1.9 Å resolution) and bulgecin·MltE (PDB ID: 4HJV, 2.3 Å resolution) complexes as guidance[2,3]. The MltE·disaccharide was in a truncated octahedral box of water molecules using the TIP3P water model, and the overall system was neutralized with 2 chlorine ions. RESP partial atomic charges were calculated for the substrate. AMBER FF99 and GAFF provided simulation parameters. The system was subjected to a 2,500-step restrained-energy minimization (on protein and substrate atoms) followed by slow heating to 300 K. The system was subjected to NPT equilibration for 250 ps with the protein and the substrate restrained. The restraints were released over 100 ps interval. Production MD was carried out at constant temperature (Langevin) and pressure (isotropic position scaling) for 20 ns using the PMEMD module of AMBER 11. The production-phase trajectory was analyzed. Several snapshots that had suitable distances ($d_3$, $d_4$, and $d_5$) for the proton transfer events (Scheme 1) were selected as the starting points for the QM/MM calculation. These snapshots were subjected to 50,000-step conjugated-gradient energy minimization, prior to selection of the suitable snapshot for calculation. All water molecules within 3 Å of the protein or within 12 Å of the QM atoms were retained. The remaining water molecules were deleted to facilitate computation. The two-layer version of the ONIOM method implemented in Gaussian 09[4] was used. The QM layer used the B3LYP/6-311++G(d,p)//B3LYP/6-31G(d) level of theory while the MM layer used the AMBER FF99 force field. The QM layer included 123 atoms: the MurNAc-GlcNAc substrate; E64; the side chains of S73, S75, and Y192; and the two active-site water molecules (Wat1 and Wat2). The electrostatic interactions and the boundary between the QM and MM regions were handled by a mechanical-embedding scheme and the link-atom approach. Mechanical-embedding scheme treats the QM/MM electronic interactions in a classical manner by using fixed atomic-point charges in the QM and MM layers. Partial atomic charges and atom types were imported from the AMBER topology for the 5214 atoms in the QM/MM model system. In the QM/MM calculations, all residues within 8 Å of the QM atoms and water molecules within 15 Å of the substrate were free to move without constraints (Supplementary Fig. 5). All other residues were fixed. Potential-energy points in the QM/MM calculations were generated over a two-dimensional grid of two direct coordinates. A QM 2D-PES scan for the boat to chair transition was carried out for the C2–C3–C4–C5 (from 63.9 to –46.1° with –5.0° interval) and H2–C2–N′–C′ (from –180.0 to 150.0° with 30.0° interval) dihedral angles at B2PLYP-D/6-311++G(d,p)//SMD M06-2X/6-31+G(d) level of theory in water. All the stationary points (species **I**, $\mathbf{Ts1_{2D}}$, $\mathbf{II_{2D}}$, $\mathbf{Ts2_{2D}}$, $\mathbf{III_{2D}}$, $\mathbf{III_{QM}}$, $\mathbf{Ts3_{QM}}$, and $\mathbf{IV_{QM}}$) were fully optimized with no reaction coordinate constraints before characterization by frequency calculations. Frequency calculations were performed with scale factors of 0.873 and 0.944 at B3LYP and M06-2X levels of theory, respectively, at 25 °C and 1 atm. The energy contours were generated using matplotlib.

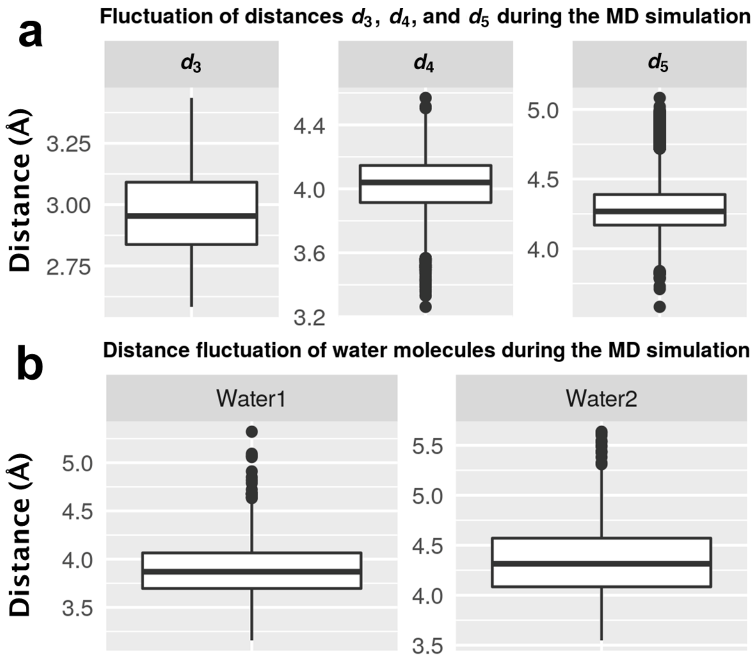


**Supplementary Figure 1.** Analysis of the MD simulation trajectory. (a) Box plot of the distribution of the distance fluctuation for $d_3$, $d_4$, and $d_5$ (black dots are outliers). (b) Distribution of the distances of the closest two water molecules to the C1 atom.

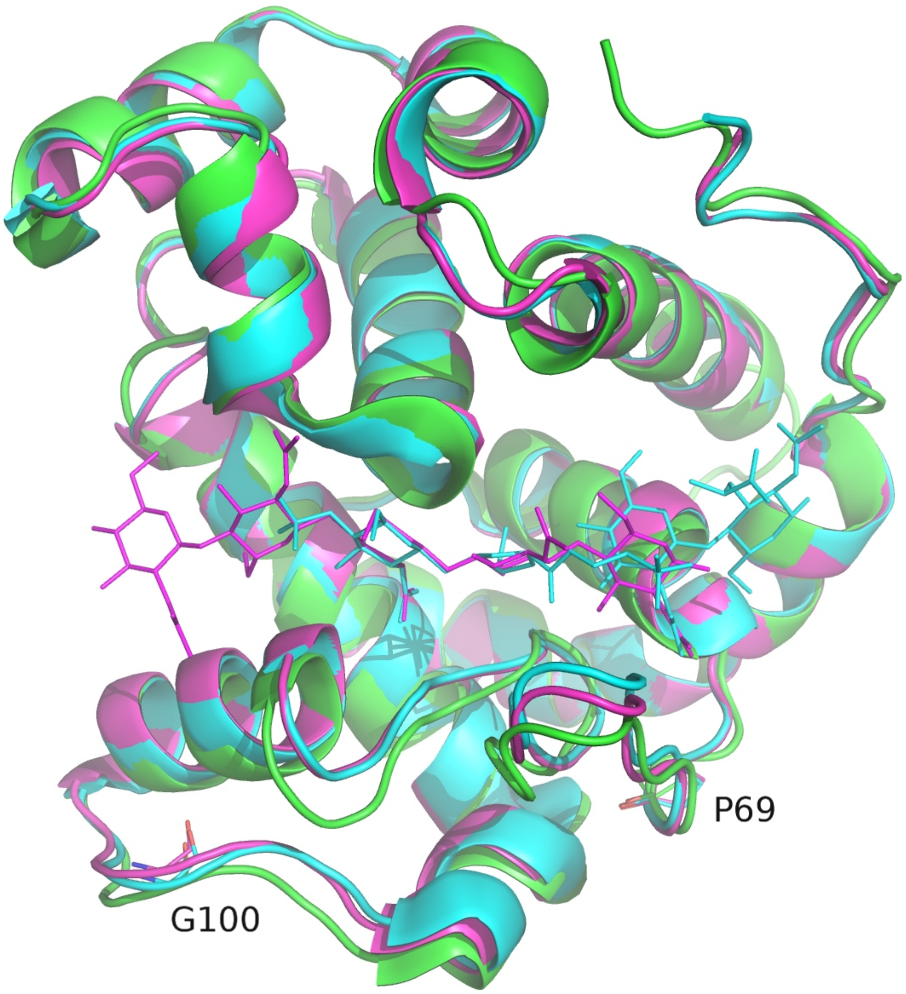


**Supplementary Figure 2.** Backbone overlay of three MltE structures (PDB ID: 2Y8P in green, PDB ID: 4HJV in cyan, and PDB ID: 4HJZ in magenta). When the apo structure (PDB ID: 2Y8P) is compared with the co-complex one with an iminosaccharide inhibitor (bulgecin) and a GlcNAc-MurNAc disaccharide (PDB ID: 4HJV), the mobile region between P69 and G100 makes a rotation of about 8° around a hinge located at P69 and G100, which in turn results in a displacement of 1.8–2.7 Å for the $C_\alpha$ atoms of the residues inside the mobile region that shape the binding site. This movement could be linked to the binding of substrate to the active site for catalysis and the release of the product.

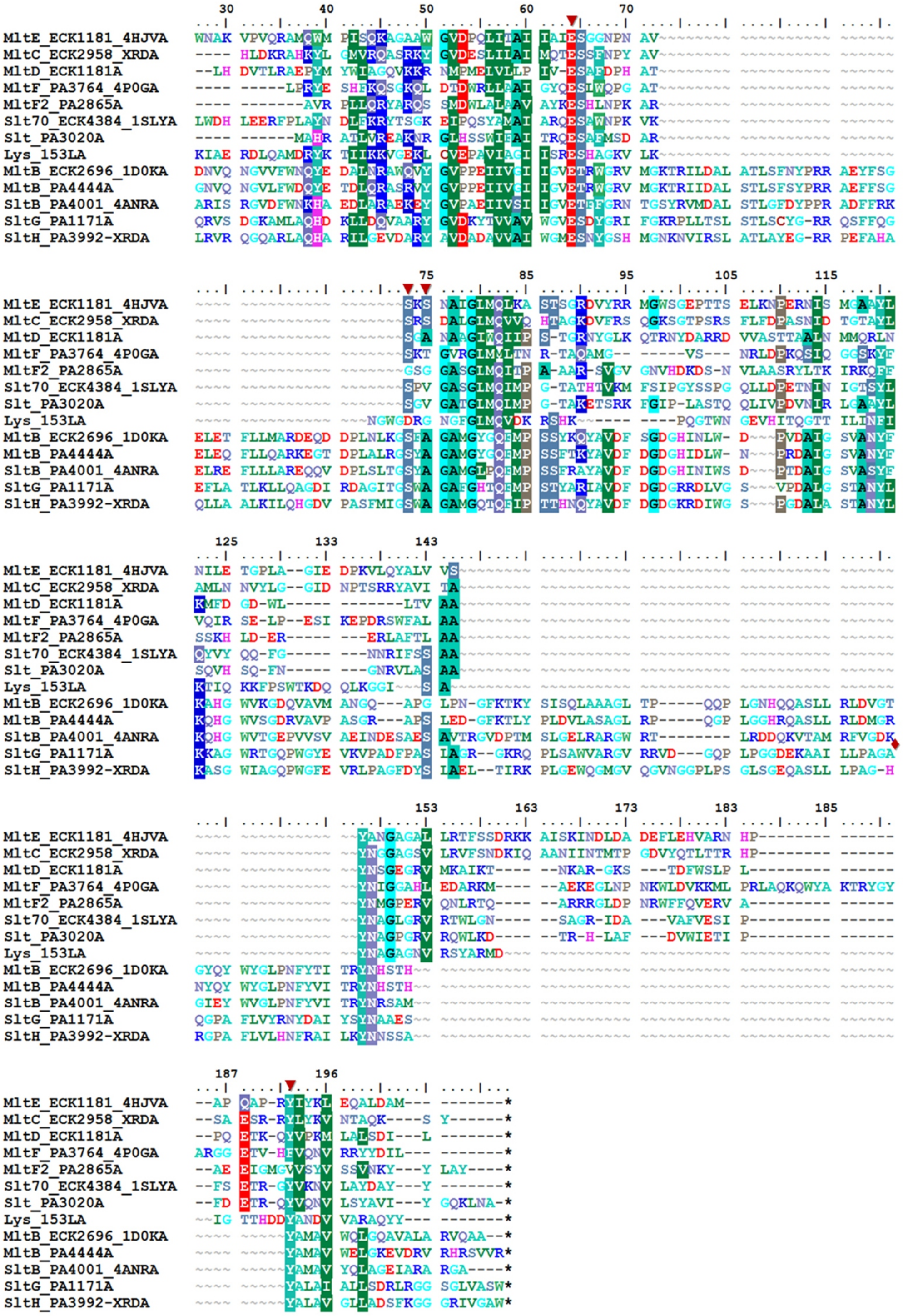


**Supplementary Figure 3.** Structure-based sequence alignment of twelve LT family members and a GH23 lysozyme. The numbering scheme is based on MltE. Residues included in the QM layer of the QM/MM calculation are marked with a red triangle on the numbering line. Along with the protein name, gene names code (NCBI) and X-ray crystal structure PDB ID codes are given when available.

## Sequence analysis of twelve LT family members and a GH23 lysozyme.

The sequence analysis (using MltE numbering) is summarized. Structure-based sequence alignment of twelve LT family members (and for reference, a GH23 lysozyme) identified sequence conservations (Supplementary Figs. 3 and 4). The catalytic glutamic acid (E64 in *E. coli* MltE) or aspartic acid is conserved in all thirteen enzymes. The subsequent residue to E64 is fully conserved as either a serine or threonine (S/T65). Among the LTs (but not the lysozyme) a serine (S73) is strongly conserved. A conserved seven-residue sequence beginning at A77 (…AIGIMQL…) is the GH23 sequence signature. The hydrophobic residues of this sequence have structural roles, perhaps with respect to positioning Q82 for substrate contact. Additional conserved residues within the LT family include a polar residue (K, R, or Q) at position 90; a hydrophobic (I or L) at position 114; a hydrophobic pair (commonly YL or YF) at positions 120-121; a fully conserved tyrosine at position 146; a conserved glycine at position 149; a highly conserved tyrosine at position 192; and a hydrophobic residue (V, L, M or I) at position 196. Additional conserved residues particular to the family 1 LTs (including MltE) are a hydrophobic residue at position 153 (L or V) and a hydrogen-bonding residue (Q in MltE, but E in the other family 1 LTs) at position 188 (see Supplementary Fig. 4 for the location of these conserved residues in the apo MltE structure (PDB ID: 2Y8P)).

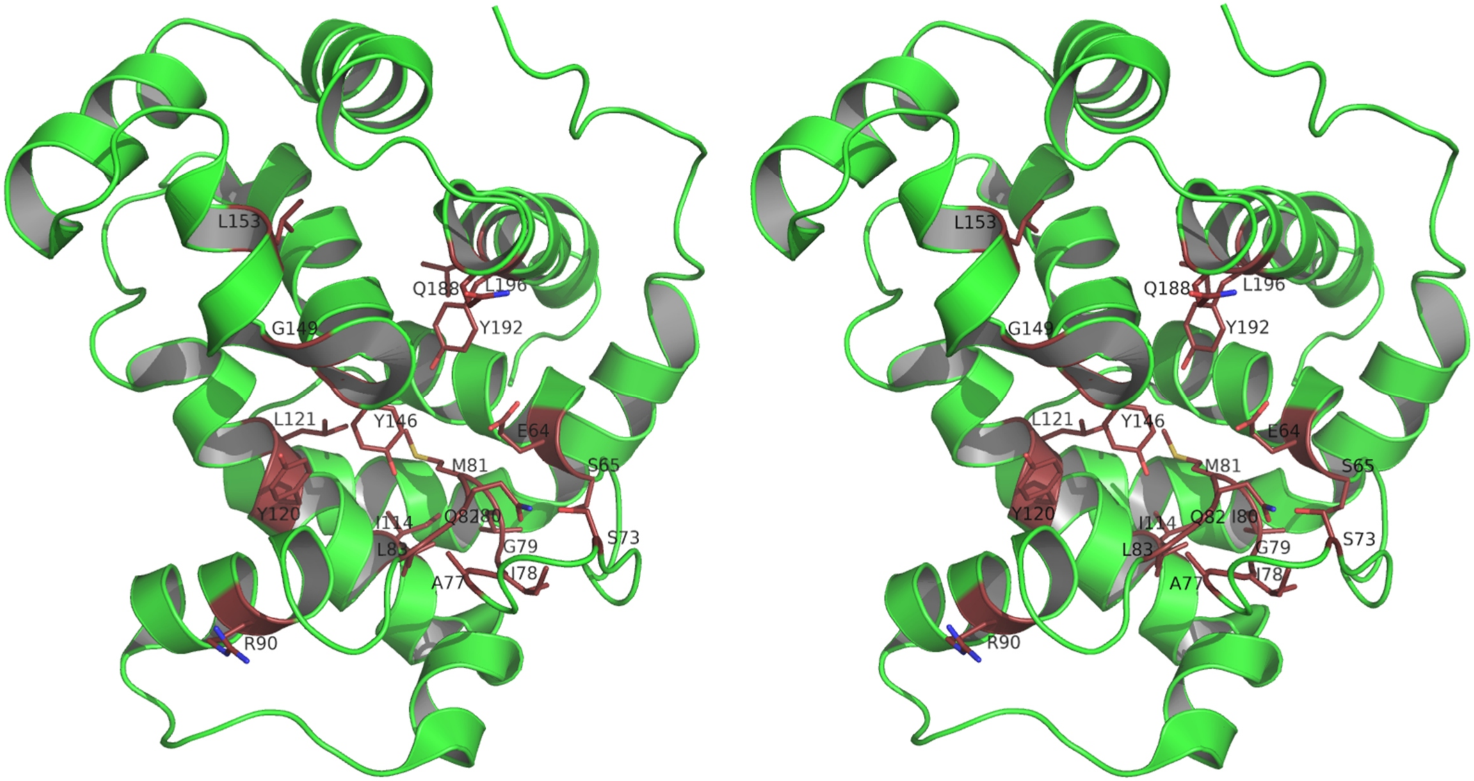


**Supplementary Figure 4.** Location of residues conserved across twelve LT family members and a GH23 lysozyme (Supplementary Fig. 3) is given in the apo structure (PDB ID: 2Y8P) of MltE as a stereo image.

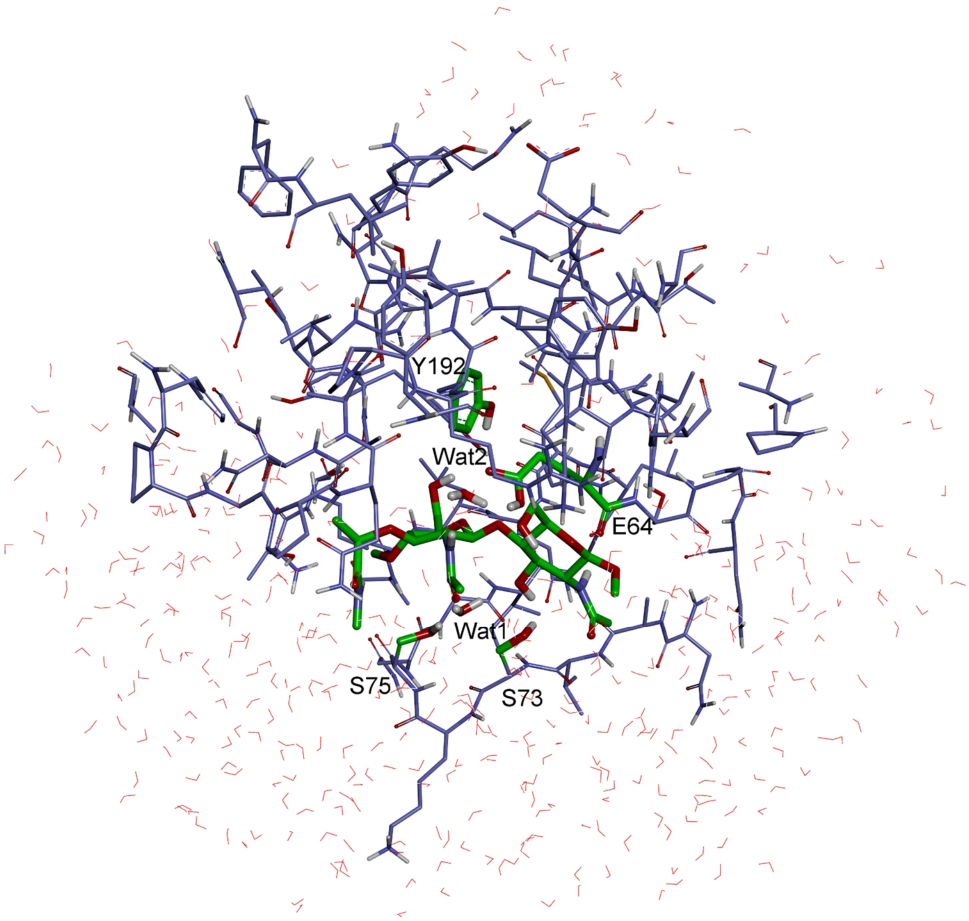


**Supplementary Figure 5.** The atoms (including the MurNAc-GlcNAc disaccharide substrate, 68 amino acid residues, and 421 water molecules) in the flexible region during geometry optimizations are shown. The QM layer represented by capped sticks includes 123 atoms: the MurNAc-GlcNAc disaccharide substrate; E64; the side chains of S73, S75, and Y192; and the two active-site water molecules (Wat1 and Wat2).

### The Michaelis complex.

The dominant features of the Michaelis complex are the hydrogen-bonding pattern of E64 and the $E_1$ conformation of the –1 MurNAc saccharide. A hydrogen bond (1.81 Å) between the $H^{\eta}$ of Y192 and the $O^{\varepsilon 1}$ of E64 orients the $H^{\varepsilon 2}$ atom of E64 into a strong hydrogen-bonding contact (1.71 Å) with the O5 of MurNAc. The $O^{\varepsilon 1}$ of E64 engages simultaneously the O6 hydrogen of the MurNAc in a second hydrogen bond (1.90 Å). A third hydrogen bond (2.48 Å) between the E64 and the glycosidic oxygen (O1) gives the foundational contact for catalysis. The simultaneous engagement of E64 with the departing oxygen of the GlcNAc, and the intercepting oxygen of the MurNAc, is enabled by the rotation of the scissile glycosidic bond. This E64 pose is appropriate to both initiate and to complete catalysis. The bound $E_1$ envelope of the –1 MurNAc is stabilized by a hydrogen bond (1.71 Å) between $H^{\varepsilon 2}$ of E64 and the O5 of MurNAc. The backbone carbonyl oxygen of E64 makes a hydrogen bond with the NH of the *N*-acetyl group of GlcNAc (2.58 Å). The two serine residues (S73 and S75) of the α3-α4 loop are proximal to the anomeric carbon of the –1 MurNAc. The $O^{\gamma}$ of S73 forms a hydrogen bond (1.87 Å) with the C3 hydroxyl hydrogen of GlcNAc. The $H^{\gamma}$ of S73 further engages in a hydrogen bond with $O^{\varepsilon 1}$ of Q82 (1.61 Å). The role of this hydrogen bond is likely stabilization of the conformation of the loop containing S73. Q82 in turn hydrogen bonds with G79. As noted previously, sequence alignment shows that G79, S73, and Q82 are highly conserved in the LT family. The hydrogen-bond network involving G79 and Q82 is likely structural. Contact of S75 with MurNAc uses a Wat1 bridge. A hydrogen atom of Wat1 also hydrogen bonds to the C4 glycosidic oxygen of MurNAc (2.04 Å). However, while S75 is conserved in a second LT (MltC) of *E. coli*, it is not conserved in other LT family members (Supplementary Fig. 3). Wat1 is in a similar position to a crystallographic water seen in MltE Q64·chitopentose (PDB ID: 4HJZ), Slt70·1,6-anhydromuropeptide (PDB ID: 1QTE), Slt35·GlcNAc (PDB ID: 1QUT), Slt35·GlcNAc-MurNAc-L-Ala-D-Glu (PDB ID: 1D0K), SltB3·GlcNAc-1,6-anhydroMurNAc-pentapeptide (PDB ID: 5AO7), and bacteriophage endolysin lambda·chitohexasaccharide (PDB IDs: 3D3D and 1D9U) complexes[2,5–9]. The oxygen atom of Wat2 hydrogen bonds (2.10 Å) to the NH of the *N*-acetyl group of MurNAc. Although Q188 is a candidate for stabilizing a potential oxazolinium intermediate (discussed later), throughout the QM/MM calculations its hydrogen bonds are to solvent (and not to substrate). The side chains of Q82 and Y146 are both distant from the substrate. Our hydrogen-bonding pattern is consistent with the X-ray structure of the MltE bulgecin A/murodipeptide co-complex[2].

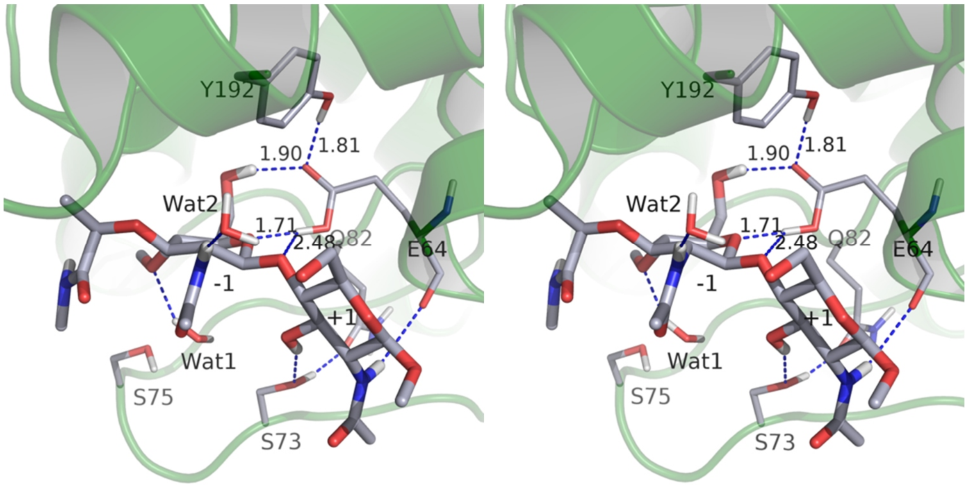


**Supplementary Figure 6.** Stereo representation of the Michaelis complex (species **I**) is given. Hydrogen bonds are represented as blue dashed lines (distances in Å). The QM layer represented by capped sticks includes 123 atoms: the MurNAc-GlcNAc disaccharide substrate; E64; the side chains of S73, S75, and Y192; and the two active-site water molecules (Wat1 and Wat2).

## One-dimensional potential-energy surface.

The competence of the Michaelis complex was tested for its ability to traverse the full reaction coordinate across a one-dimensional (1D) potential-energy surface (PES) scan. The 1D-PES scan was conducted for critical distance $d_2$ (initially the glycosidic bond, lengthening during catalysis: Scheme 1) from 1.4 to 3.0 Å. Unfortunately, a steric clash between the departing GlcNAc and the protein occurred for $d_2$ >2.8 Å. As an alternative, the PES is obtained using the geometric parameter distance $d_1$ (the distance between the anomeric C1 atom of MurNAc and the C4 atom of GlcNAc). Interestingly, progression of the Michaelis complex (**I**) to 1,6-anhydroMurNAc in a $B_{3,O}$ conformation occurs spontaneously during the $d_1$ scan. The progressive increase in $d_1$, which ultimately breaks the glycosidic bond, suffices for progression to products. The system is poised for the reaction outcome.

### Characterization of species $II_{2D}$.

Intermediates having a $^{2,5}B$ conformation of species $\mathbf{II_{2D}}$ were seen previously for the retaining GH11 xylanase[10,11], the GH39 alpha-L-iduronidase[12], the inverting GH6 cellulases[13,14], and the GH8 endoglucanase CelA[15]. During the progression of **I** to $\mathbf{II_{2D}}$, both the *N*-acetyl carbonyl oxygen of MurNAc and the MurNAc O6 move closer to the anomeric carbon (C1). The corresponding distances, $d_4$ and $d_5$, decrease by 0.08 Å (from 3.22 to 3.14 Å) and 0.93 Å (from 4.17 to 3.24 Å), respectively (Scheme 1). The proximity of these oxygen atoms to the anomeric C1 atom stabilizes the transient development of the positive charge on C1 that results from the lengthening of glycosidic bond. These interactions may account for the location on this surface of species $\mathbf{II_{2D}}$ as a local energy minimum. Its location at $d_3$ = 1.50 Å indicates a strong hydrogen bond between the glycosidic oxygen and the $H^{\varepsilon 2}$ of E64. The value for $d_2$ at this minimum (1.80 Å) indicates a lengthened, but not yet broken, glycosidic bond (Supplementary Fig. 7). Natural atomic orbital-based Wiberg bond indices (which represent a type of "bond order" between atoms)[16] were calculated for the MurNAc-GlcNAc disaccharide substrate (in the absence of protein) at QM B3LYP/6-311++G(d,p) level of theory and used to compare the bond strengths for the glycosidic bond in species $\mathbf{II_{2D}}$ compared to the Michaelis complex (**I**). This analysis shows that the glycosidic bond in species $\mathbf{II_{2D}}$ has a partial-bond character of 0.677 (compared to 0.921 for the Michaelis complex). Species with partial-bonding character at the scissile glycosidic bond have been reported previously in the literature[17,18].

The value for the out-of-plane angle ($\theta_{H1}$) for atom H1 of species $\mathbf{II_{2D}}$ (with respect to the plane defined by O5, C1, and C2) is 41.3°. This value indicates that H1 (the hydrogen atom on the anomeric C1 carbon) lacks the flatness of a discrete oxocarbenium species. We compared the energy of species $\mathbf{II_{2D}}$ with a modeled species (in the absence of protein) in which the proton transfer from E64 to the glycosidic oxygen was complete ($d_2$ = 1.80 Å, $d_3$ = 1.00 Å). Single-point calculations at QM B3LYP/6-311++G(d,p) level of theory with the conductor-like polarizable continuum model (CPCM)[19,20] in water shows that species $\mathbf{II_{2D}}$ is more stable by 6.05 kcal·mol$^{-1}$ than the species in which the proton has completely transferred. This result indicates that species $\mathbf{II_{2D}}$ is not the artifact of QM/MM calculations, and supports the partial-bonding character at the glycosidic bond of species $\mathbf{II_{2D}}$. The proton of the glutamic acid transfers completely to the glycosidic oxygen at $d_2$ = 2.00 Å.

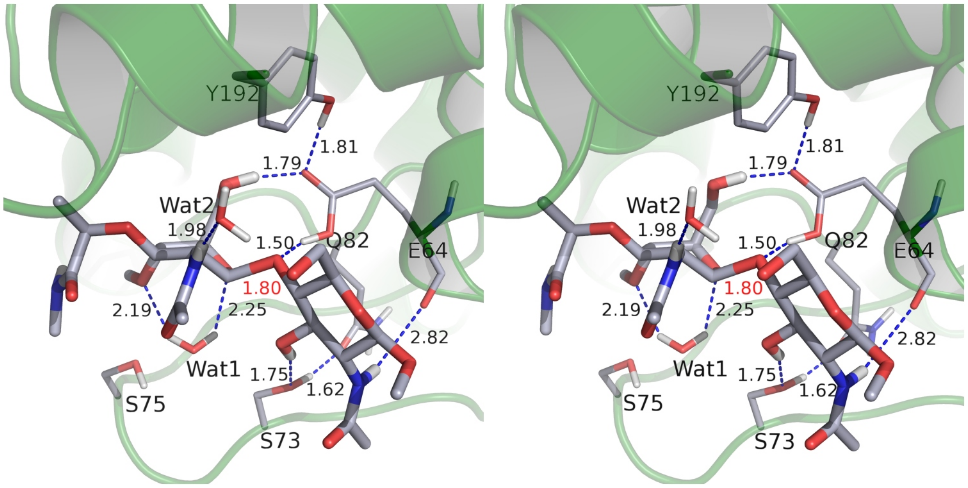


**Supplementary Figure 7.** Stereo representation of the intermediate (species $\mathbf{II_{2D}}$) is given. Hydrogen bonds are represented as blue dashed lines (distances in Å). The distance between the C1 carbon of MurNAc and the glycosidic oxygen is shown in red. The QM layer of 123 atoms is shown using capped stick representation: the MurNAc-GlcNAc disaccharide substrate; E64; the side chains of S73, S75, and Y192; and the two active-site water molecules (Wat1 and Wat2).

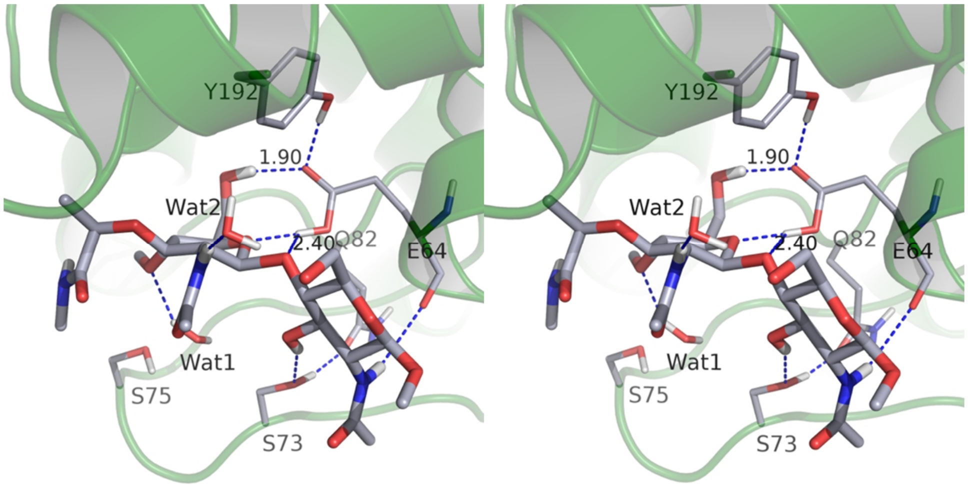


**Supplementary Figure 8.** Stereo representation of transition species (**Ts1$_{2D}$**) between the Michaelis complex (species **I**) and the intermediate (species **II$_{2D}$**) is given. Hydrogen bonds are represented as blue dashed lines (distances in Å). The QM layer of 123 atoms is shown using capped stick representation: the MurNAc-GlcNAc disaccharide substrate; E64; the side chains of S73, S75, and Y192; and the two active-site water molecules (Wat1 and Wat2).

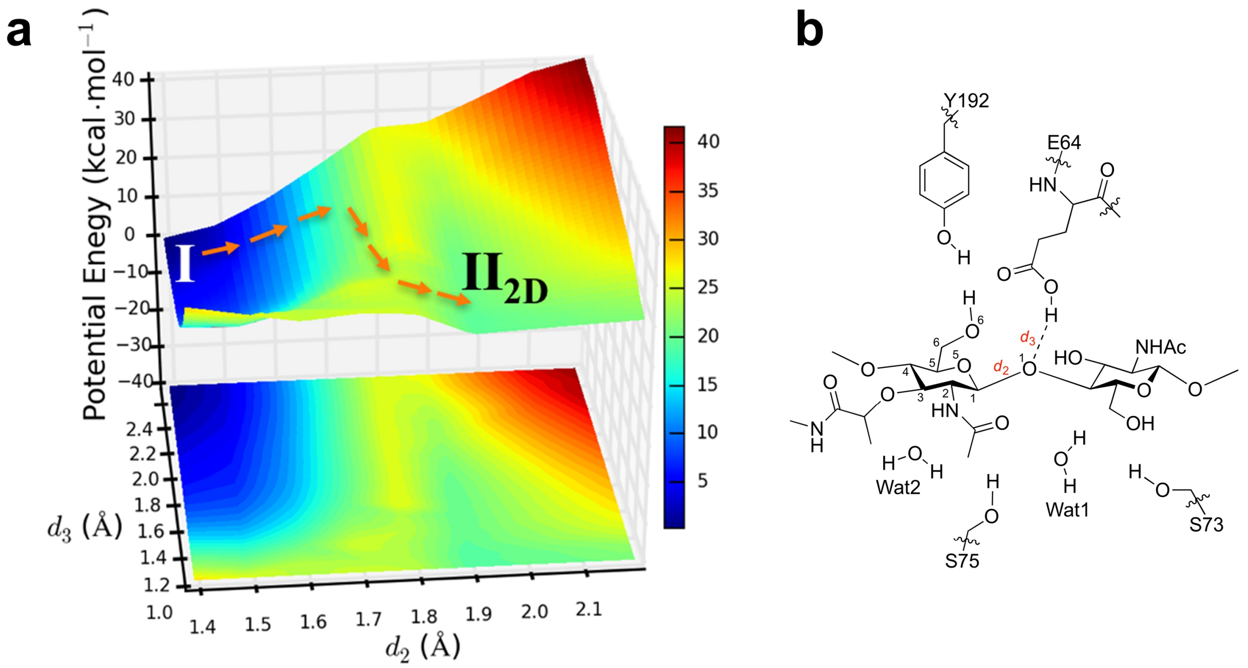


**Supplementary Figure 9.** (a) QM/MM potential-energy surface with respect to the $d_2$ (scanned from 1.39 to 2.19 Å at 0.10 Å interval and $d_3$ (scanned from 2.50 to 1.00 Å at –0.10 Å interval) reaction coordinates, with Wat1 deleted from the QM layer. The rate-limiting reaction path from the Michaelis complex (**I**) to the intermediate (**II$_{2D}$**) is shown with orange arrows. (b) Schematic view of the $d_2$ and $d_3$ reaction coordinates.

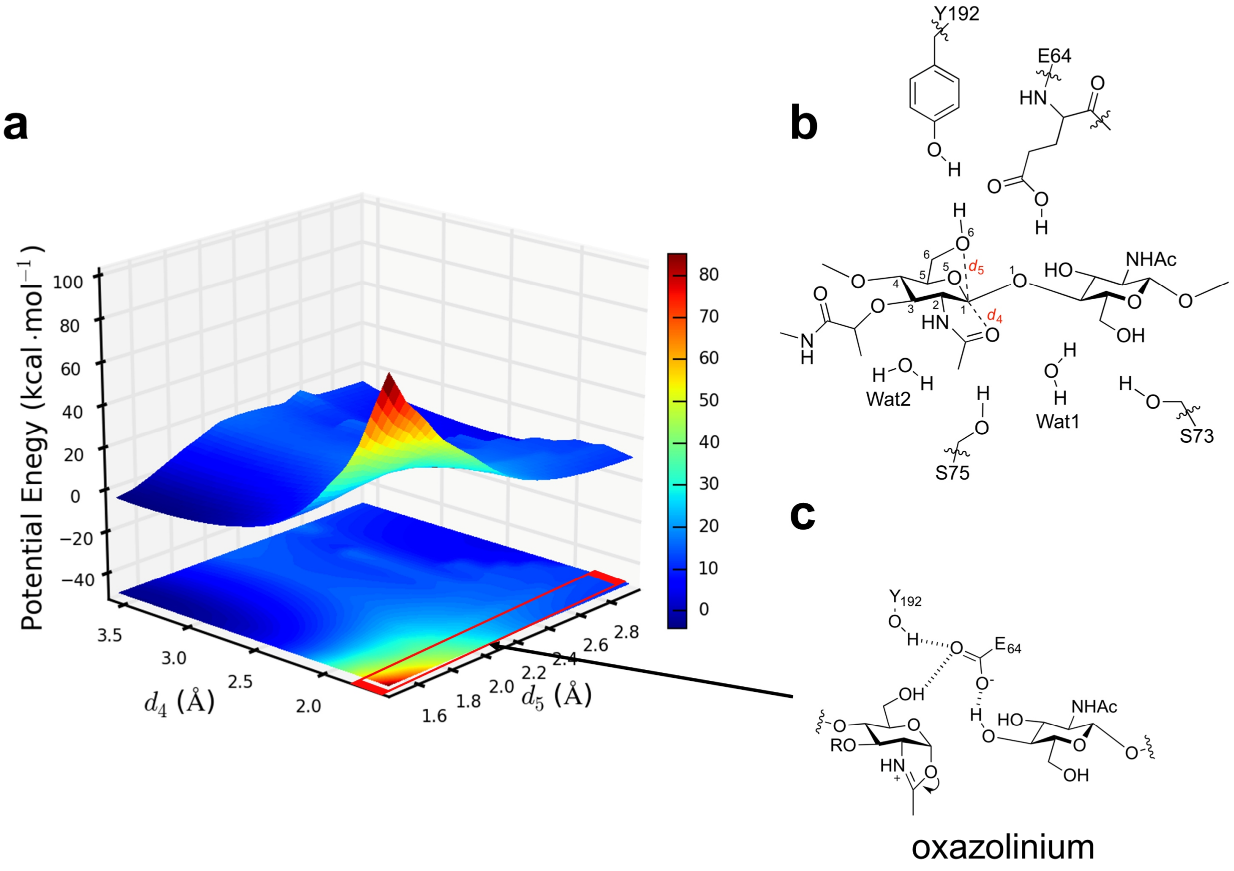


**Supplementary Figure 10.** (a) Two-dimensional potential-energy surface with respect to the $d_4$ (scanned from 1.58 to 3.58 Å at 0.10 Å interval) and $d_5$ (scanned from 2.96 to 1.46 Å at –0.10 Å interval) reaction coordinates. The region in red rectangular box indicates oxazolinium species. (b) Schematic view of the $d_4$ and $d_5$ reaction coordinates. (c) Chemical structure of the oxazolinium species.

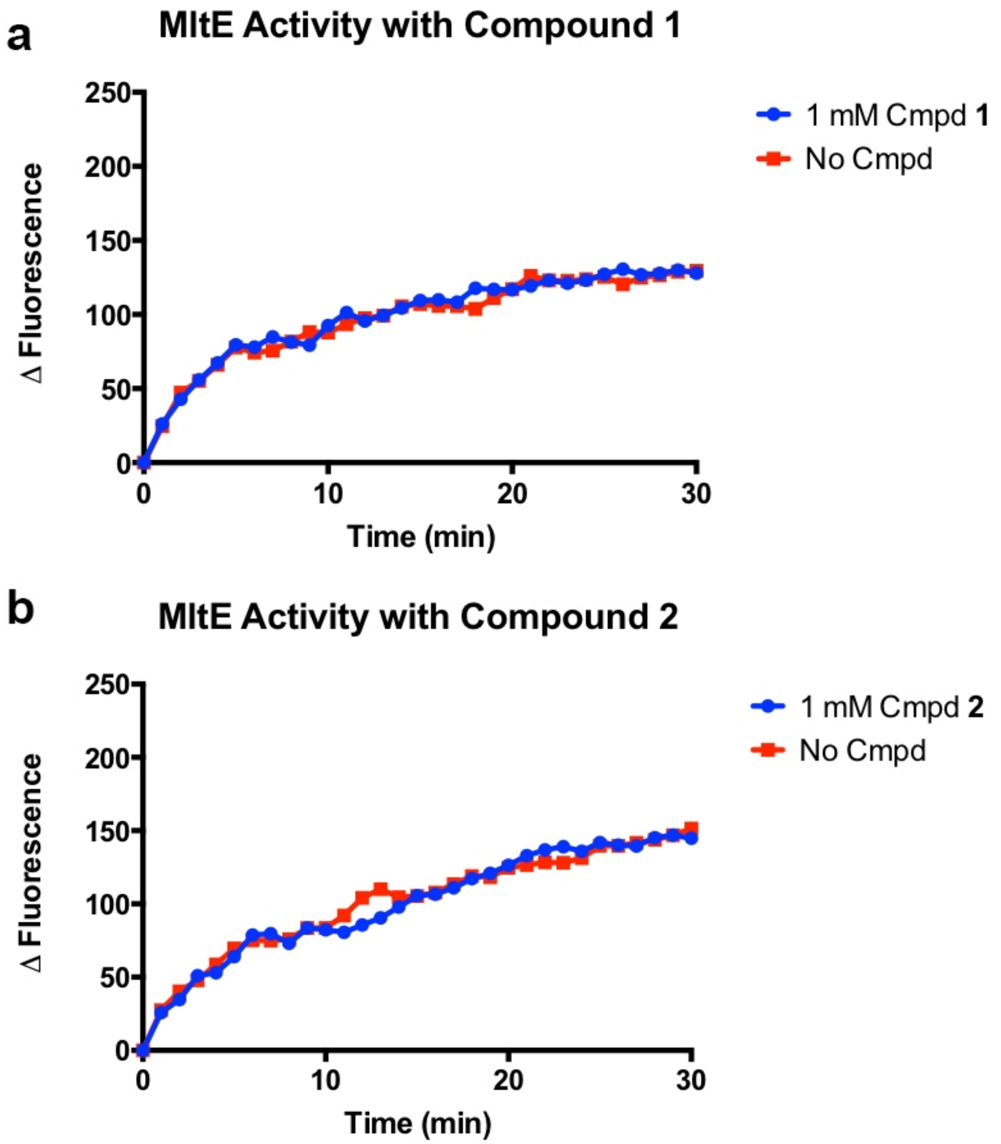


**Supplementary Figure 11.** The *E. coli* MltE fluorescence activity assays in the presence of 1 mM compounds **1** and **2**.

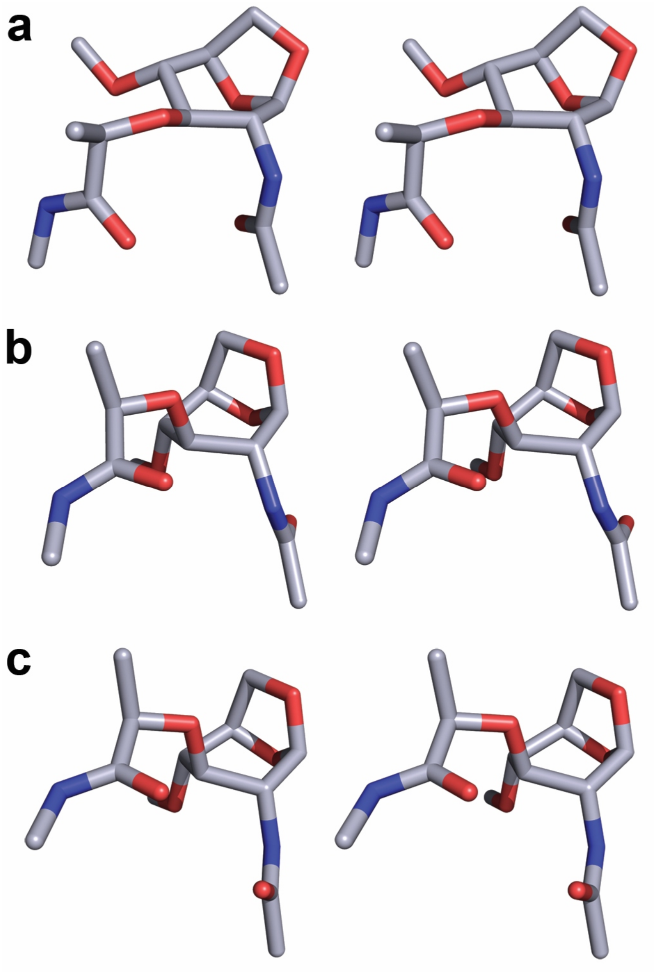

**Supplementary Figure 12.** (a) Stereo representation of the 1,6-anhydroMurNAc (**III$_{QM}$**) in the $B_{3,O}$ conformation; (b) the transition species (**Ts3$_{QM}$**, $E_O$ conformation) between species **III$_{QM}$** and **IV$_{QM}$**; (c) the 1,6-anhydroMurNAc (**IV$_{QM}$**) in the $^1C_4$ conformation is given. A progressive viewing from the top to the bottom in stereo will show how the transformation takes place.

**Supplementary Table 1.** Selected Natural Population Atomic Charges, Distances, and Angles of Reaction Species.[a]

| | **I** | **Ts1**$_{\mathbf{2D}}$ | **II**$_{\mathbf{2D}}$ | **Ts2**$_{\mathbf{2D}}$ | **III**$_{\mathbf{2D}}$ | **Ts3**$_{\mathbf{QM}}$ | **IV**$_{\mathbf{QM}}$ |
|---|---|---|---|---|---|---|---|
| Charges (*e*) | | | | | | | |
| C1 | 0.434 | 0.449 | 0.451 | 0.522 | 0.420 | 0.439 | 0.442 |
| C2 | –0.059 | –0.083 | –0.110 | –0.144 | –0.065 | –0.073 | –0.083 |
| O1 | –0.600 | –0.626 | –0.684 | –0.749 | –0.731 | NA | NA |
| O5 | –0.622 | –0.609 | –0.553 | –0.440 | –0.571 | –0.565 | –0.604 |
| O6 | –0.730 | –0.729 | –0.737 | –0.700 | –0.627 | –0.604 | –0.592 |
| $O_{NAc}$[b] | –0.638 | –0.636 | –0.634 | –0.619 | –0.639 | –0.635 | –0.635 |
| $N_{NAc}$[b] | –0.637 | –0.635 | –0.635 | –0.622 | –0.632 | –0.631 | –0.639 |
| H1 | 0.200 | 0.212 | 0.216 | 0.255 | 0.223 | 0.199 | 0.188 |
| Distances (Å) | | | | | | | |
| C1–O5 | 1.44 | 1.40 | 1.35 | 1.28 | 1.40 | 1.41 | 1.41 |
| C1–C2 | 1.54 | 1.52 | 1.52 | 1.51 | 1.55 | 1.54 | 1.53 |
| C1–O1 ($d_2$) | 1.40 | 1.70 | 1.80 | 2.90 | 2.97 | NA | NA |
| O6–C1 ($d_5$) | 4.17 | 4.09 | 3.24 | 2.29 | 1.49 | 1.43 | 1.42 |
| $O_{NAc}$[b]–C1 ($d_4$) | 3.22 | 3.22 | 3.14 | 2.83 | 3.50 | 2.96 | 4.19 |
| $O^{\varepsilon1}$[c]–C1 | 4.46 | 4.45 | 4.56 | 4.19 | 3.86 | NA | NA |
| $O^{\varepsilon2}$[c]–C1 | 3.49 | 3.56 | 3.84 | 4.02 | 3.77 | NA | NA |
| Angles (°) | | | | | | | |
| C5–O5–C1–C2 | –73.6 | –74.7 | 2.9 | 37.4 | 74.0 | 72.4 | 77.0 |
| $\theta_{H1}$[d] | 52.9 | 41.3 | 39.4 | 10.9 | 49.0 | 49.1 | 52.5 |

[a]The natural population atomic charges are calculated for the MurNAc (or 1,6-anhydroMurNAc)-GlcNAc (**I**, **Ts1**$_{\mathbf{2D}}$, **II**$_{\mathbf{2D}}$, **Ts2**$_{\mathbf{2D}}$, and **III**$_{\mathbf{2D}}$) and the 1,6-anhydroMurNAc (**Ts3**$_{\mathbf{QM}}$ and **IV**$_{\mathbf{QM}}$) at B3LYP/6-311++G(d,p) level of theory in the absence of protein, whereas distances, C5–O5–C1–C2 dihedral angles, and $\theta_{H1}$ out-of-plane angles are obtained by B3LYP/6-31G(d):AMBER (**I**, **Ts1**$_{\mathbf{2D}}$, **II**$_{\mathbf{2D}}$, **Ts2**$_{\mathbf{2D}}$, and **III**$_{\mathbf{2D}}$) and SMD M06-2X/6-31+G(d) (**Ts3**$_{\mathbf{QM}}$ and **IV**$_{\mathbf{QM}}$) optimizations. [b]$O_{NAc}$ and $N_{NAc}$ describes the *N*-acetyl carboxyl oxygen and the *N*-acetyl nitrogen of MurNAc or 1,6-anhydroMurNAc, respectively. [c]$O^{\varepsilon1}$ and $O^{\varepsilon2}$ denote the carboxylate oxygen atoms of E64. [d]$\theta_{H1}$ is the out-of-plane angle for atom H1 (the hydrogen atom on the anomeric C1 carbon) with respect to the plane defined by atoms O5, C1, and C2 of –1 MurNAc.

## Synthesis of compounds

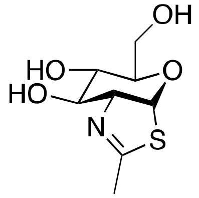


NAG-thiazoline **3** was synthesized as previously described[21].

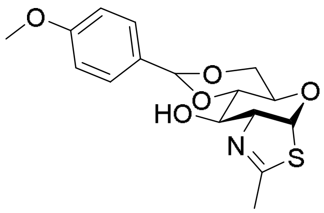


**(3a*R*,4a*R*,8a*S*,9*R*,9a*R*)-7-(4-Methoxyphenyl)-2-methyl-3a,4a,5,8a,9,9a-hexahydro-[1,3]dioxino[4',5':5,6]pyrano[3,2-*d*]thiazol-9-ol (4).**

The synthesis of the titled compound was similar to the reported route of Wong and Ritter[22]. NAG-thiazoline **3** (1.5 g, 7.1 mmol) was dissolved in anhydrous dimethylformamide (36 mL) and *p*-methoxylbenzaldehyde dimethyl acetal (1.4 mL, 8.2 mmol) and *p*-toluenesulfonic acid monohydrate (137 mg, 0.7 mmol) were added. The reaction mixture was heated for 4 H at 50 °C. The solvent was removed by rotary evaporation and the mixture was purified by silica chromatography (100% ethyl acetate) to yield 1.8 g (77%) of the titled compound as a white powder. TLC (100% ethyl acetate): 0.37 $R_f$. $^1$H NMR (500 MHz, $CDCl_3$) δ ppm 2.30 (s, 3 H) 3.49 (br. s, 1 H) 3.59 (t, *J* = 9.4 Hz, 1 H) 3.75 (t, *J* = 10.5 Hz, 1 H) 3.82 (s, 3 H) 3.82-3.88 (m, 1 H) 3.96 (td, *J* = 9.7, 5.1 Hz, 1 H) 4.26-4.40 (m, 2 H) 5.56 (s, 1 H) 6.55 (d, *J* = 7.6 Hz, 1 H) 6.79-6.97 (m, 2 H) 7.34-7.49 (m, 2 H). $^{13}$C NMR (101 MHz, $CDCl_3$) δ ppm 22.29, 55.33, 64.90, 68.19, 73.53, 75.88, 77.23, 78.71, 93.15, 102.00, 113.69, 127.63, 129.41, 160.29. HRMS (m/z): $[M + H]^+$, calcd for $C_{16}H_{20}NO_5S$, 338.1057; found, 338.1037.

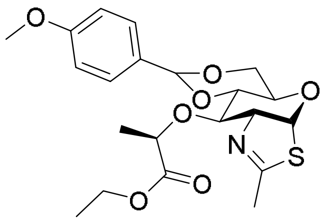


**Ethyl (2*R*)-2-(((3a*R*,4a*R*,8a*S*,9*R*,9a*R*)-7-(4-methoxyphenyl)-2-methyl-3a,4a,5,8a,9,9a-hexahydro-[1,3]dioxino[4',5':5,6]pyrano[3,2-*d*]thiazol-9-yl)oxy)propanoate (5).**

Compound **4** (1.05 g, 3.1 mmol) was dissolved in anhydrous dichloromethane (20 mL) and NaH (100 mg, 95%, 4 mmol) was added and the mixture was allowed to stir for 15 min at RT. Another portion of NaH was added (4 mmol) followed by another 15 min stir period. (*S*)-Lactic triflate ethyl ester[23] (1.2 mL, 6.2 mmol) was added dropwise and the solution was allowed to stir for 15 min. Another portion of (*S*)-lactic triflate ethyl ester (0.6 mL, 3.1 mmol) was added and the solution was stirred for an additional 10 min. The reaction mixture was diluted with dichloromethane and a couple drops of water was added to the solution, which was concentrated by rotary evaporation. The mixture was purified by silica chromatography (3:7 ethyl acetate:hexanes to 2:3 ethyl acetate:hexanes) to yield 1.0 g (73%) of the titled compound as a waxy solid. TLC (1:1 ethyl acetate:hexanes): 0.47 $R_f$. $^1$H NMR (400 MHz, $CDCl_3$) δ ppm 1.26 (t, *J* = 7.1 Hz, 3 H) 1.42 (d, *J* = 6.9 Hz, 3 H) 2.24 (d, *J* = 1.7 Hz, 3 H) 3.63-3.75 (m, 2 H) 3.75-3.79 (m, 1 H) 3.79-3.84 (m, 3 H) 3.94 (dd, *J* = 7.6, 5.9 Hz, 1 H) 4.13-4.23 (m, 2 H) 4.28 (dd, *J* = 10.3, 4.7 Hz, 1 H) 4.34 (q, *J* = 6.9 Hz, 1 H) 4.42-4.50 (m, 1 H) 5.54 (s, 1 H) 6.41 (d, *J* = 7.3 Hz, 1 H) 6.78-6.93 (m, 2 H) 7.33-7.48 (m, 2 H). $^{13}$C NMR (101 MHz, $CDCl_3$) δ ppm 14.23, 18.98, 21.92, 55.29, 60.79, 64.40, 68.67, 75.62, 77.54, 80.08, 80.14, 92.45, 101.58, 113.58, 127.33, 129.68, 160.05, 167.38, 173.01. HRMS (m/z): $[M + H]^+$, calcd for $C_{21}H_{28}NO_7S$, 438.1581; found, 438.1578.

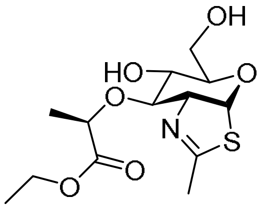

**Ethyl (*R*)-2-(((3a*R*,5*R*,6*S*,7*R*,7a*R*)-6-hydroxy-5-(hydroxymethyl)-2-methyl-3a,6,7,7a-tetrahydro-5H-pyrano[3,2-*d*]thiazol-7-yl)oxy)propanoate (6).**

Similar to the route of Wong and Ritter[22], compound **5** (0.2 g, 0.46 mmol) was dissolved in acetonitrile (5 mL) and *p*-toluenesulfonic acid monohydrate (92 mg, 0.48 mmol) and ethanedithiol (43 μL, 0.51 mmol) were added and the solution was stirred overnight at RT. The solution was concentrated by rotary evaporation and the mixture was purified by silica chromatography (1:9 methanol: dichloromethane) to yield 115 mg (78%) of the titled compound as an oil. TLC (1:9 methanol: dichloromethane): 0.47 $R_f$. $^1$H NMR (400 MHz, $CD_3OD$) δ ppm 1.30 (t, *J* = 7.2 Hz, 3 H) 1.39 (d, *J* = 6.9 Hz, 3 H) 2.27 (d, *J* = 2.2 Hz, 3 H) 3.12-3.23 (m, 1 H) 3.56 (dd, *J* = 12.1, 6.2 Hz, 1 H) 3.64-3.78 (m, 2 H) 4.09 (dd, *J* = 3.6, 1.8 Hz, 1 H) 4.22 (qd, *J* = 7.1, 1.6 Hz, 2 H) 4.34 (q, *J* = 7.0 Hz, 1 H) 4.57 (dtd, *J* = 5.8, 2.3, 2.3, 1.1 Hz, 1 H) 6.31 (d, *J* = 7.1 Hz, 1 H). $^{13}$C NMR (101 MHz, $CD_3OD$) δ ppm 13.14, 17.93, 18.96, 60.80, 62.22, 68.03, 73.85, 73.92, 77.71, 79.90, 88.84, 169.34, 173.62. HRMS (m/z): $[M + H]^+$, calcd for $C_{13}H_{22}NO_6S$, 320.1162; found, 320.1163.

NMRS for **(3a*R*,4a*R*,8a*S*,9*R*,9a*R*)-7-(4-methoxyphenyl)-2-methyl-3a,4a,5,8a,9,9a-hexahydro-[1,3]dioxino[4',5':5,6]pyrano[3,2-*d*]thiazol-9-ol** (**4**)

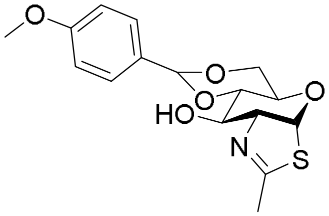


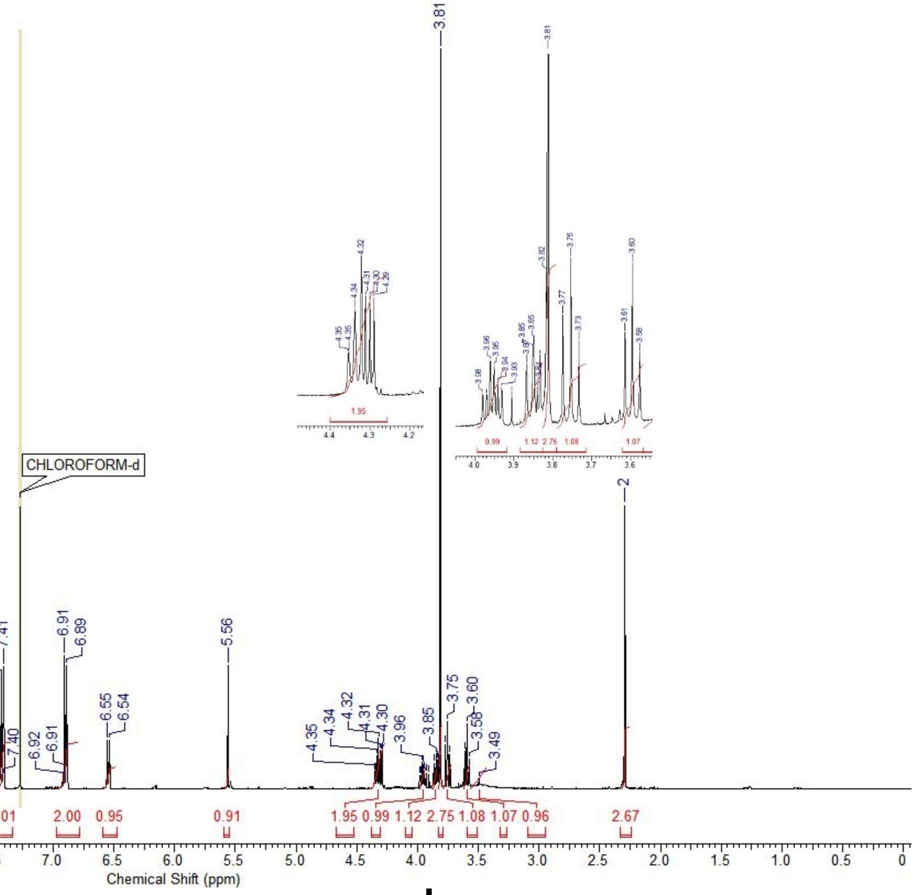


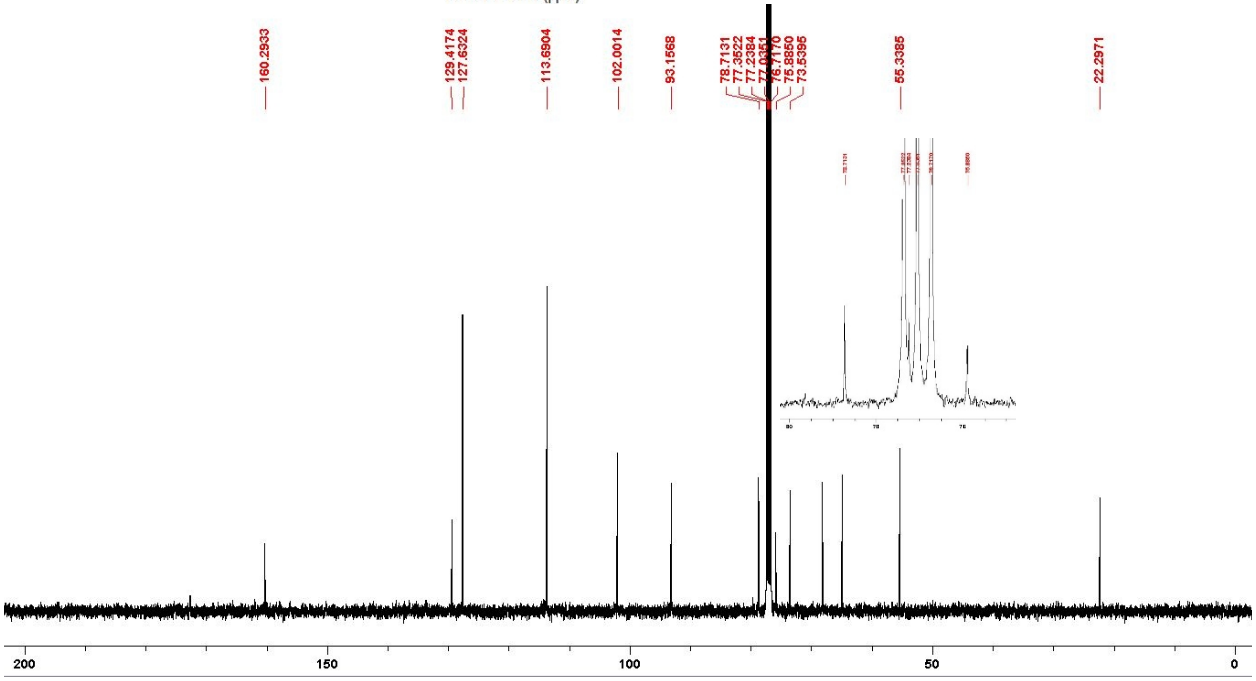

NMRS for **ethyl (2*R*)-2-(((3a*R*,4a*R*,8a*S*,9*R*,9a*R*)-7-(4-methoxyphenyl)-2-methyl-3a,4a,5,8a,9,9a-hexahydro-[1,3]dioxino[4',5':5,6]pyrano[3,2-*d*]thiazol-9-yl)oxy)propanoate** (**5**)

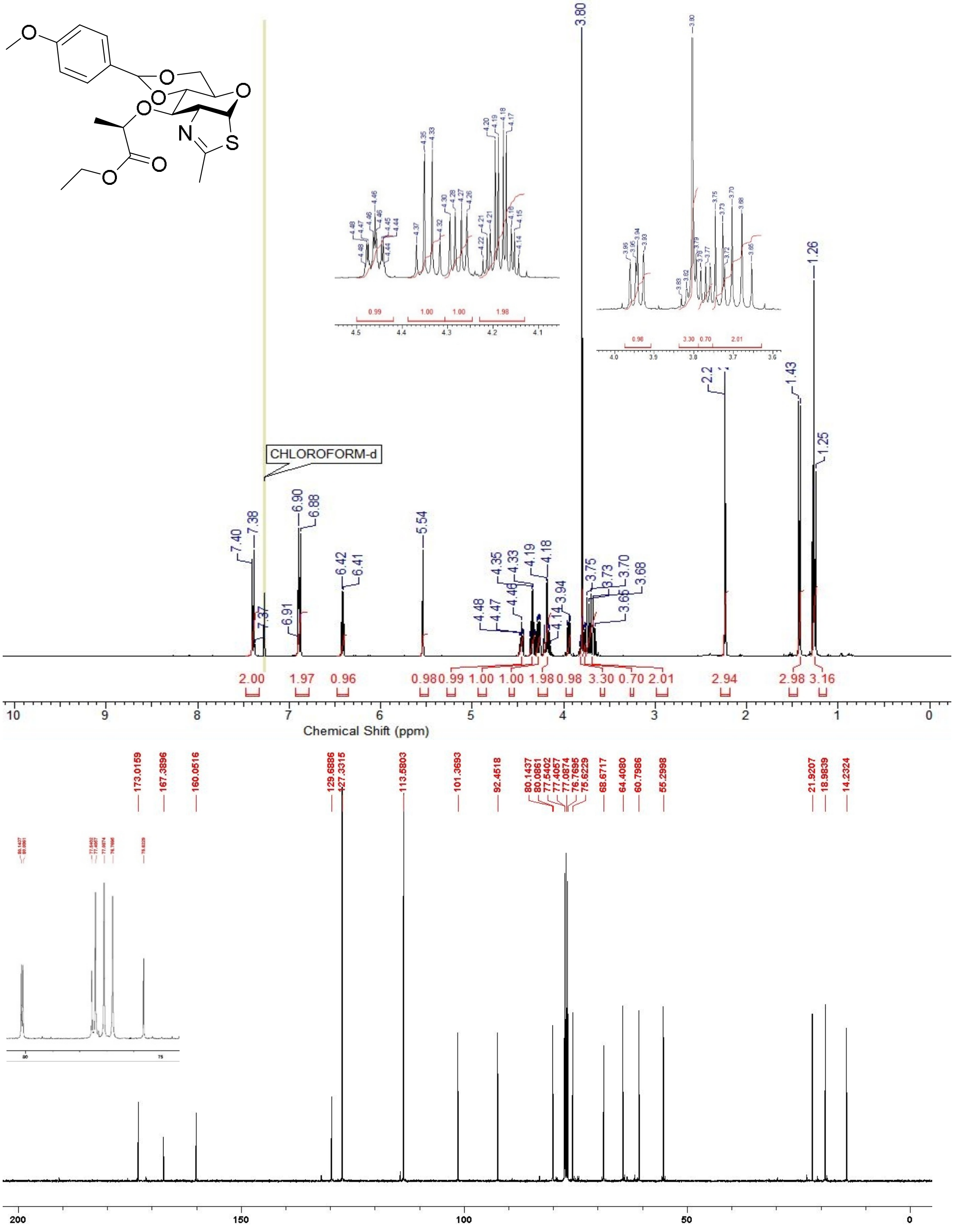

NMRS for **ethyl (*R*)-2-(((3a*R*,5*R*,6*S*,7*R*,7a*R*)-6-hydroxy-5-(hydroxymethyl)-2-methyl-3a,6,7,7a-tetrahydro-5H-pyrano[3,2-*d*]thiazol-7-yl)oxy)propanoate** (**6**)

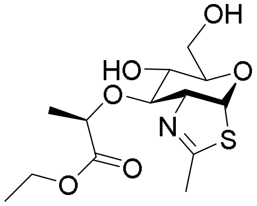


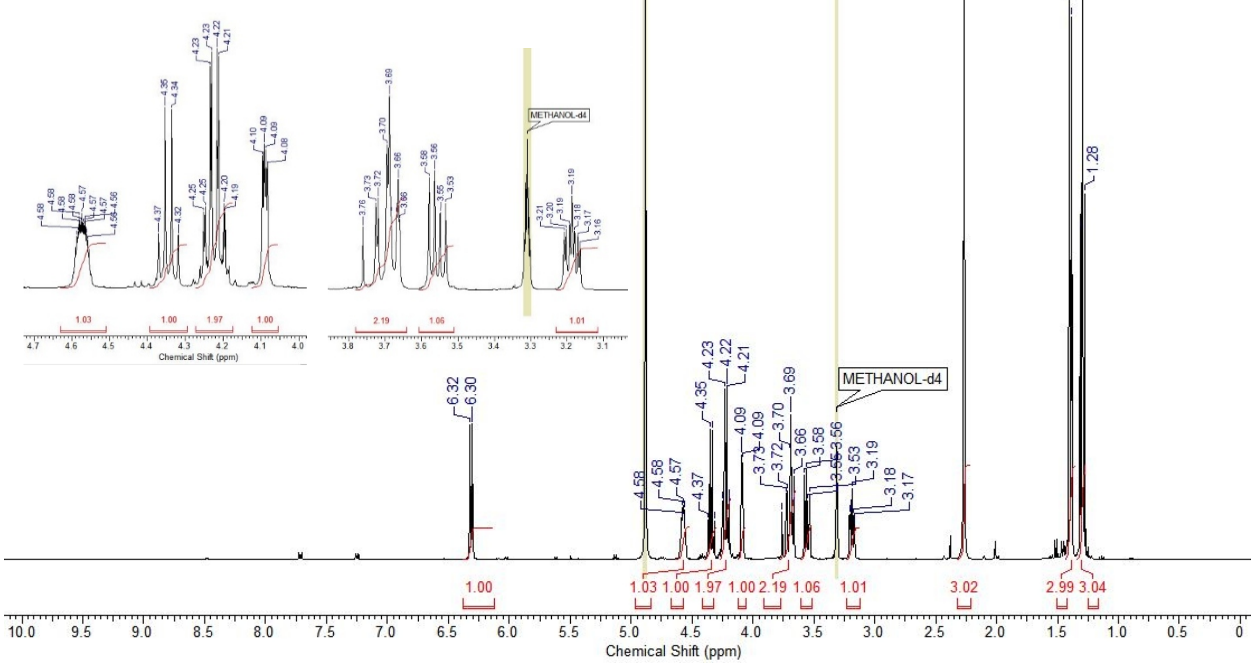


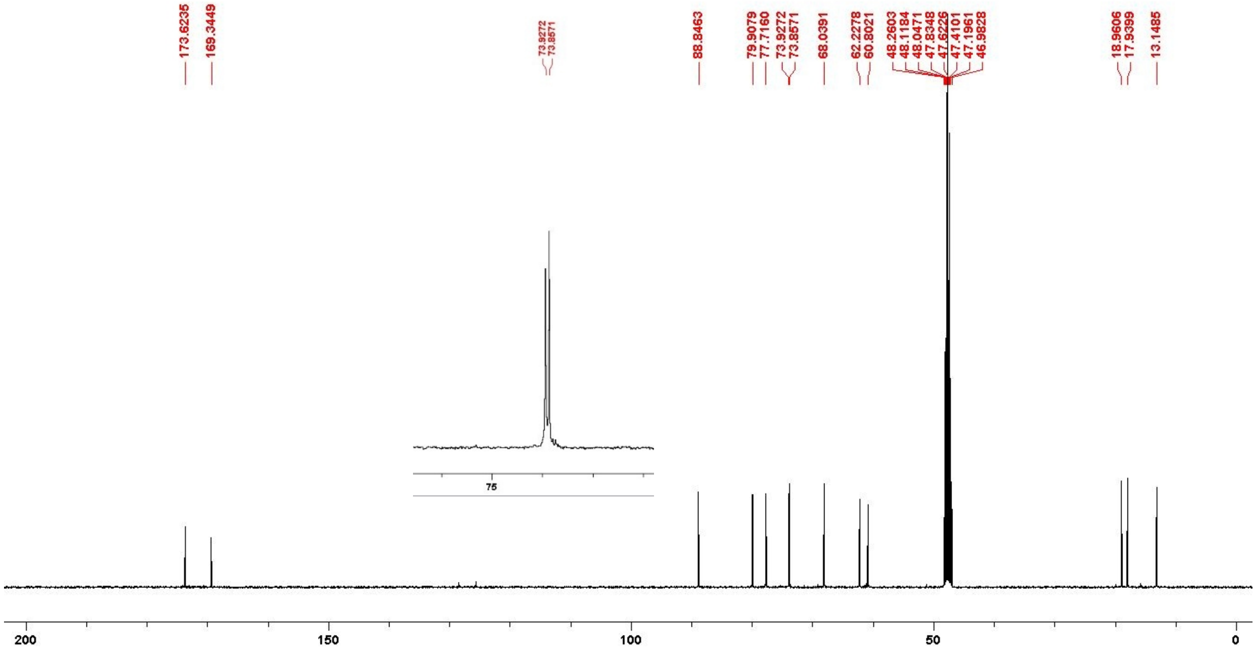

NMRS for **lithium (*R*)-2-(((3a*R*,5*R*,6*S*,7*R*,7a*R*)-6-hydroxy-5-(hydroxymethyl)-2-methyl-3a,6,7,7a-tetrahydro-5H-pyrano[3,2-*d*]thiazol-7-yl)oxy)propanoate** (**2**)

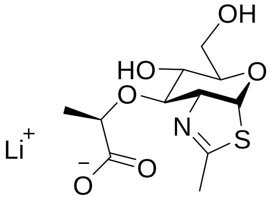


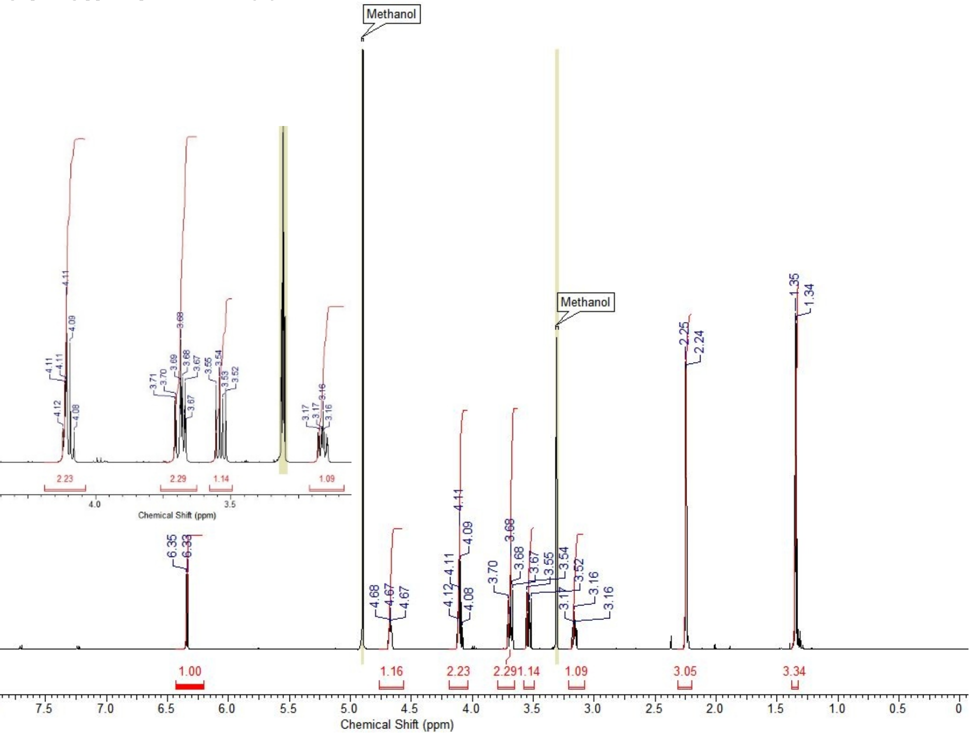


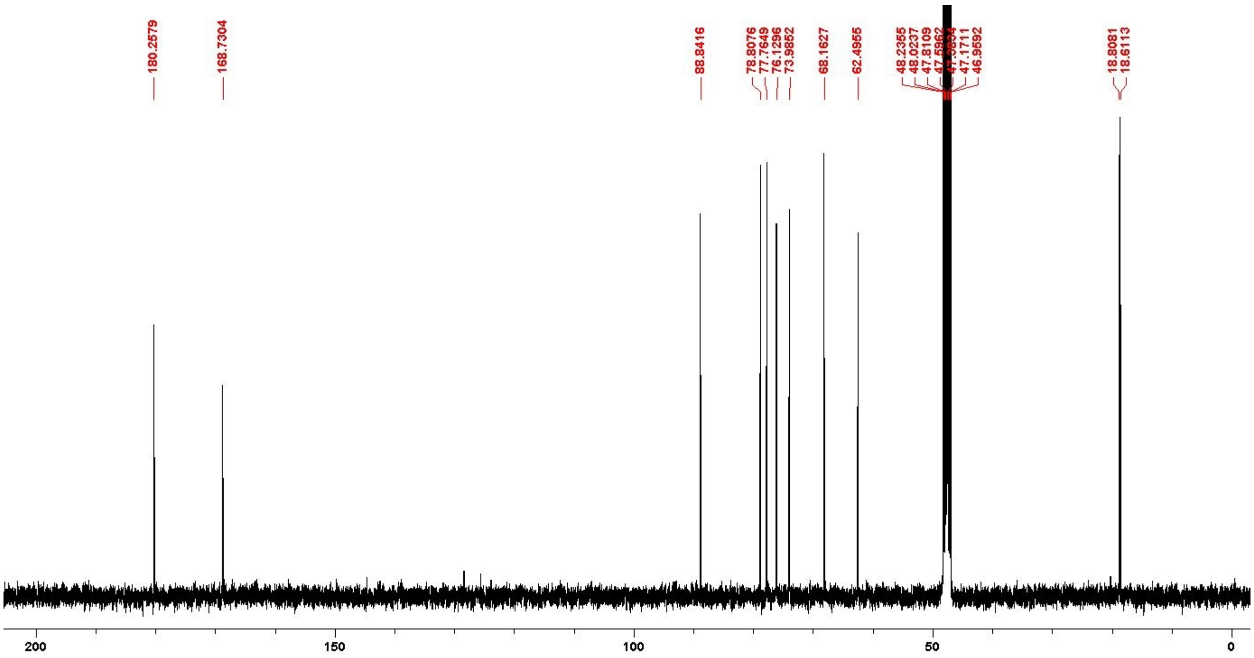

NMRS for **(*R*)-2-(((3a*R*,5*R*,6*S*,7*R*,7a*R*)-6-hydroxy-5-(hydroxymethyl)-2-methyl-3a,6,7,7a-tetrahydro-5H-pyrano[3,2-d]thiazol-7-yl)oxy)propanamide** (**1**)

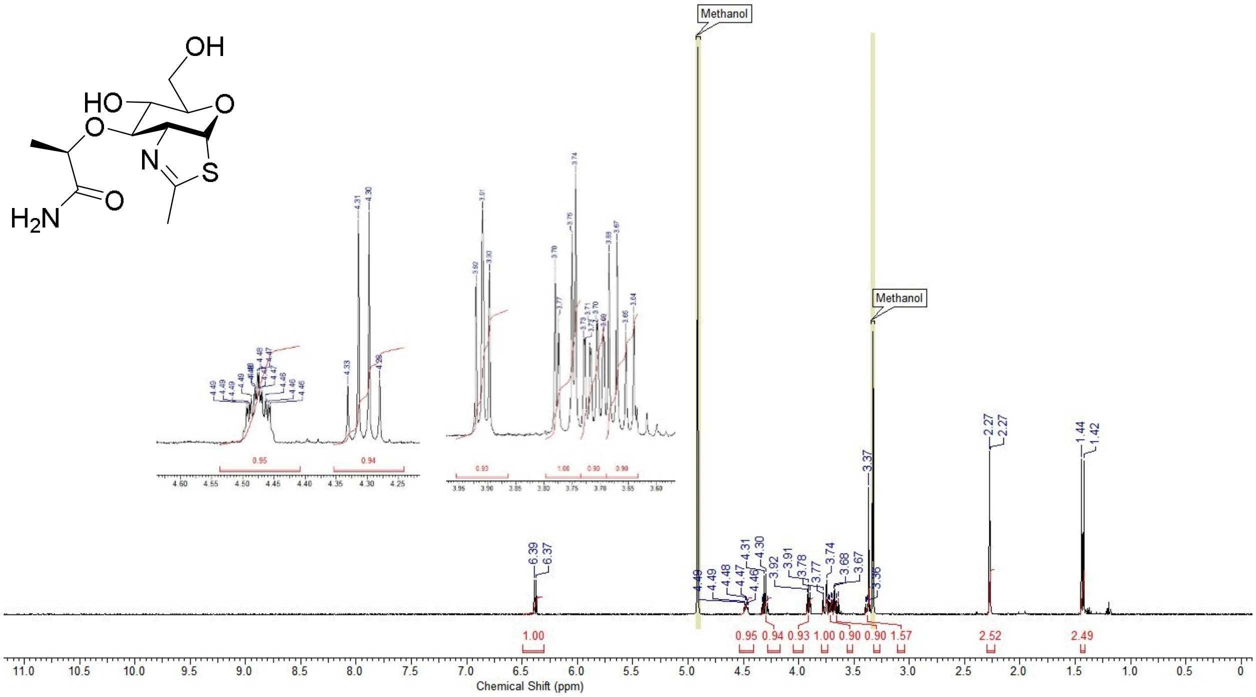


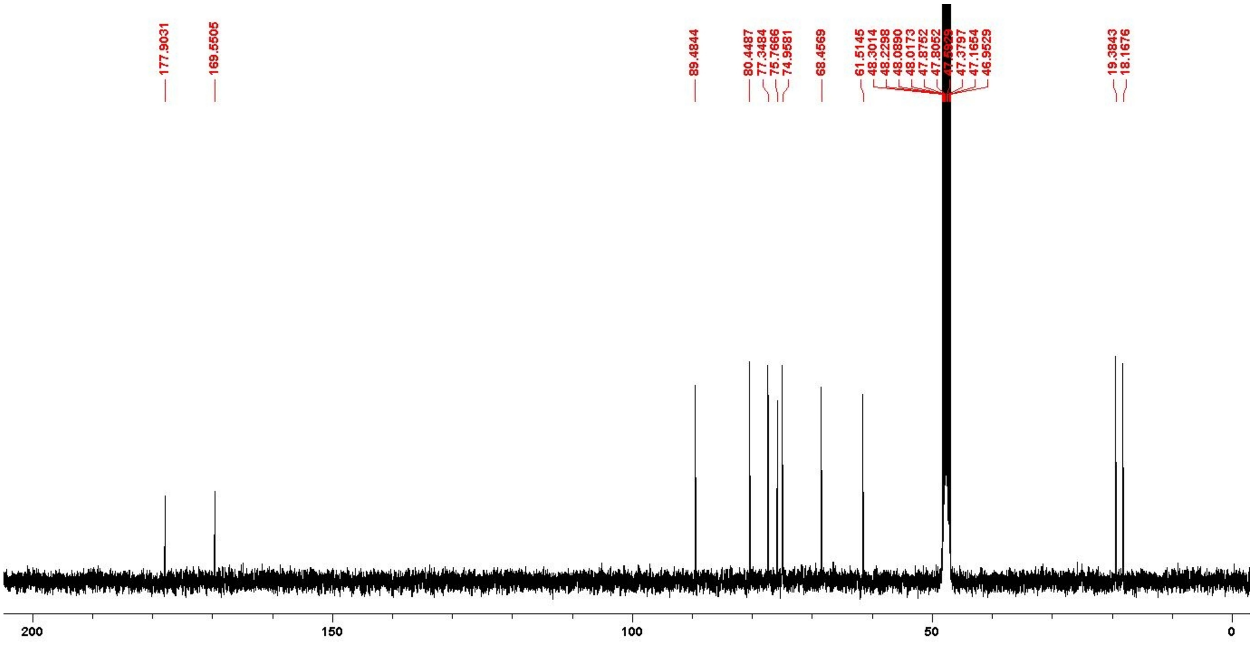

## References


1. Case, D. A. *et al.* *AMBER11*; University of California; San Francisco, 2010.
2. Fibriansah, G., Gliubich, F. I. & Thunnissen, A.-M. W. H. On the mechanism of peptidoglycan binding and cleavage by the endo-specific lytic transglycosylase MltE from *Escherichia coli*. *Biochemistry* **51**, 9164–9177 (2012).
3. Artola-Recolons, C. *et al.* High-resolution crystal structure of MltE, an outer membrane-anchored endolytic peptidoglycan lytic transglycosylase from *Escherichia coli*. *Biochemistry* **50**, 2384–2386 (2011).
4. Frisch, M. J. *et al. Gaussian 09*; Revision D.01; Gaussian, Inc.; Wallingford, CT, 2013.
5. van Asselt, E. J., Thunnissen, A.-M. W. H. & Dijkstra, B. W. High resolution crystal structures of the *Escherichia coli* lytic transglycosylase Slt70 and its complex with a peptidoglycan fragment. *J. Mol. Biol.* **291**, 877–898 (1999).
6. van Asselt, E. J. *et al.* Crystal structure of *Escherichia coli* lytic transglycosylase Slt35 reveals a lysozyme-like catalytic domain with an EF-hand. *Structure* **7**, 1167–1180 (1999).
7. van Asselt, E. J., Kalk, K. H. & Dijkstra, B. W. Crystallographic studies of the interactions of *Escherichia coli* lytic transglycosylase Slt35 with peptidoglycan. *Biochemistry* **39**, 1924–1934 (2000).
8. Lee, M. *et al.* Turnover of bacterial cell wall by SltB3, a multidomain lytic transglycosylase of *Pseudomonas aeruginosa*. *ACS Chem. Biol.* **11**, 1525–1531 (2016).
9. Leung, A. K.-W., Duewel, H. S., Honek, J. F. & Berghuis, A. M. Crystal structure of the lytic transglycosylase from bacteriophage lambda in complex with hexa-*N*-acetylchitohexaose. *Biochemistry* **40**, 5665–5673 (2001).
10. Sabini, E. *et al.* Catalysis and specificity in enzymatic glycoside hydrolysis: a $^{2,5}B$ conformation for the glycosyl-enzyme intermediate revealed by the structure of the *Bacillus agaradhaerens* family 11 xylanase. *Chem. Biol.* **6**, 483–492 (1999).
11. Sidhu, G. *et al.* Sugar ring distortion in the glycosyl-enzyme intermediate of a family G/11 xylanase. *Biochemistry* **38**, 5346–5354 (1999).
12. Bie, H. *et al.* Insights into mucopolysaccharidosis I from the structure and action of α-L-iduronidase. *Nat. Chem. Biol.* **9**, 739–745 (2013).
13. Varrot, A. *et al.* Distortion of a cellobio-derived isofagomine highlights the potential conformational itinerary of inverting β-glucosidases. *Chem. Commun.* 946–947 (2003).
14. Varrot, A. *et al. Mycobacterium tuberculosis* strains possess functional cellulases. *J. Biol. Chem.* **280**, 20181–20184 (2005).
15. Guérin, D. M. A. *et al.* Atomic (0.94 Å) resolution structure of an inverting glycosidase in complex with substrate. *J. Mol. Biol.* **316**, 1061–1069 (2002).
16. Wiberg, K. B. Application of the pople-santry-segal CNDO method to the cyclopropylcarbinyl and cyclobutyl cation and to bicyclobutane. *Tetrahedron* **24**, 1083–1096 (1968).
17. Smith, B. J. A conformational study of 2-oxanol: insight into the role of ring distortion on enzyme-catalyzed glycosidic bond cleavage. *J. Am. Chem. Soc.* **119**, 2699–2706 (1997).
18. Tvaroska, I., André, I. & Carver, J. P. Ab Initio molecular orbital study of the catalytic mechanism of glycosyltransferases: description of reaction pathways and determination of transition-state structures for inverting *N*-acetylglucosaminyltransferases. *J. Am. Chem. Soc.* **122**, 8762–8776 (2000).
19. Barone, V. & Cossi, M. Quantum calculation of molecular energies and energy gradients in solution by a conductor solvent model. *J. Phys. Chem. A* **102**, 1995–2001 (1998).
20. Cossi, M., Rega, N., Scalmani, G. & Barone, V. Energies, structures, and electronic properties of molecules in solution with the C-PCM solvation model. *J. Comput. Chem.* **24**, 669–681 (2003).
21. Knapp, S. *et al.* NAG-thiazoline, an *N*-acetyl-β-hexosaminidase inhibitor that implicates acetamido participation. *J. Am. Chem. Soc.* **118**, 6804–6805 (1996).
22. Ritter, T. K. & Wong, C.-H. Synthesis of *N*-acetylglucosamine thiazoline/lipid II hybrids. *Tetrahedron Lett.* **42**, 615–618 (2001).
23. Kawasaki, A. *et al.* Synthesis of diaminopimelic acid containing peptidoglycan fragments and tracheal cytotoxin (TCT) and investigation of their biological functions. *Chem. Eur. J.* **14**, 10318–10330 (2008).
